# Plasma Concentration of 12-Hydroxyeicosatetraenoic Acid, Single Nucleotide Polymorphisms of 12-Lipooxygenase Gene and Vaso-Occlusion in Sickle Cell Disease

**DOI:** 10.3389/fgeed.2021.722190

**Published:** 2021-08-26

**Authors:** Augustine Nwakuche Duru, Sunday Ocheni, Obike Ibegbulam, Iheanyi Okpala

**Affiliations:** Department of Haematology and Immunology, University of Nigeria College of Medicine, Ituku-Ozalla, Nigeria

**Keywords:** sickle, vaso-occlusion, inflammaion, 12-hydroxyeicosatetraenoic acid, 12-lipoxygenase, ALOX12 gene polymorphism

## Abstract

**Background and Novel Aspect of this Work:** In the light of previous findings that inflammation predisposes to intercellular adhesion and microvascular occlusion in sickle cell disease (SCD), this study investigated the relationship between the number of vaso-occlusive events in SCD, plasma levels of the pro-inflammatory molecules 12-Hydroxyeicosatetraenoic acid (12-HETE)**,** TNF-α and IL-1β; and single nucleotide polymorphisms (SNPs) in the gene 12-Lipooxygenase (ALOX-12), which encodes the enzyme 12-Lipoxygenase that catalyzes the biosynthesis of 12-HETE.

**Objective:** To evaluate the relationship between vaso-occlusion in SCD and plasma concentrations of 12-HETE, TNF-α, and IL-1β; and single nucleotide polymorphisms (SNPs) in ALOX-12 gene.

**Participants and Methods:** In 50 HbSS patients, the numbers of vaso-occlusive crisis requiring hospital treatment in the previous 1 year and the vaso-occlusive complications of SCD developed to date (e.g stroke) were added to obtain the vaso-occlusive events (VOE) score. In the HbSS patients and 30 healthy sibling control persons, plasma concentrations of 12-HETE, TNF-α and IL-1β were measured by ELISA, the ALOX12 SNPs rs2073438 and rs1126667 detected by DNA sequencing, and the accrued data statistically analyzed.

**Results:** Compared to SCD patients with VOE score 0–1, those with scores ≥3 had higher plasma levels of 12-HETE (*p* < 0.0001) and TNF-α (*p* = 0.19), but not IL-1β (*p* = 0.27). VOE score showed strong direct correlation with plasma level of 12-HETE (r = 0.65, *p* < 0.0001), but not with TNF-α nor IL-1β. Neither VOE score nor plasma concentration of 12-HETE showed any relationship with the ALOX12 SNPs rs2073438 and rs1126667.

**Conclusion:** The strong direct correlation of VOE score with plasma concentration of 12-HETE suggests that the clinical relevance of this pro-inflammatory molecule in SCD-associated vaso-occlusion needs to be evaluated in further studies.

## Introduction

Sickle cell disease (SCD) is the most common inherited blood condition in humans. (Davies and Brozovic, 1989; Sergeant, 1997; Escoffery and Suzanne, 1998; Wheatherall and Clegg, 2001; Okpala, 2004a). Approximately 300,000 children are born annually with SCD worldwide. (Okpala, 2004a). This blood disorder is associated with continual ischaemic tissue injury and inflammatory reaction. (Hedo et al., 1993; Platt, 2000; Conran and Belcher, 2018; Piccin et al., 2019; Sundd et al., 2019; Nader et al., 2020). Vascular endothelium is activated at the sites of inflammation and leucocytes secrete pro-inflammatory molecules such as tumour necrosis factor-alpha (TNF-α), interleukin-1β (IL-1β) and interleukin 8 (IL-8). (Belcher et al., 2000). Activated endothelial cells increase their expression of the ligands for adhesion molecules on leucocytes, erythrocytes and platelets; such as selectins, intercellular adhesion molecule-1 (ICAM-1) and vascular cell adhesion molecule-1 (VCAM-1). (Carlos and Harlan, 1994; Springer, 1994; Osarogiagbon, 2001). Evidence for inflammation in SCD includes raised leucocyte count, plasma levels of TNF-α, IL-1β, IL-8 and platelet activating factor. (Kaul et al., 1989; Francis and Haywood, 1992; Kaul et al., 1996; Okpala, 2004b; Frenette, 2004; Okpala, 2006). The pro-inflammatory molecule 12-hydroxyeicosaenoic acid (12-HETE) is a member of the leukotriene biosynthesis pathway produced in the presence of oxygen by the catalytic action of the enzyme 12-Lipooxygenase on arachidonic acid (AA). (Setty et al., 1998; Micheal et al., 2009). Adherence of erythrocytes, leucocytes and platelets to other blood cells and the vascular endothelium contributes to microvascular occlusion in SCD. (Kaul et al., 1989; Francis and Haywood, 1992; Kaul et al., 1996; Okpala, 2004b; Frenette, 2004; Okpala, 2006). The formation of such heterocellular aggregates is mediated by intercellular adhesion molecules, including selectins, ICAM-1 and VCAM-1. By stimulating greater expression of adhesion molecules on blood and vascular endothelial cells, inflammation increases the risk of vaso-occlusive events (VOE) in SCD. (Francis and Haywood, 1992; Okpala, 2004b). Previous research by Setty and co-investigators showed that, relative to control individuals, plasma 12-HETE levels are increased in SCD patients in steady state, rising further during vaso-occlusive crises; and that this pro-inflammatory molecule enhances both basal and hypoxia-induced expression of vascular-endothelial cell adhesion molecule (VICAM-1) which mediates sickle erythrocyte adherence to blood vessel endothelium (Setty et al., 1998). These findings suggest that 12-HETE could have a role in the pathogenesis of SCD-associated vaso-occlusion. The relationship between the number of vaso-occlusive events in SCD and plasma concentration of the pro-inflammatory molecule 12-HETE, or single nucleotide polymorphisms of the gene that codes for its biosynthetic enzyme (12-Lipooxygenase), has not been previously investigated.

The aim of this study was to evaluate the relationship between the number of vaso-occlusive events in SCD, plasma concentrations of the pro-inflammatory molecules 12-HETE, TNF-α, and IL-1β, and the SNPs rs2073438 and rs1126667 (Gln261Arg) in ALOX12 gene which encodes the enzyme 12-Lipooxygenase that catalyses the biosynthesis of 12-HETE. The effects of these two ALOX12 SNPs on plasma 12-HETE and the number of vaso-occlusive events in SCD were evaluated because rs2073438 had been associated with high urinary concentration of 12-HETE in a previous study (Quintana et al., 2006); and rs1126667 is one of the only two common missense SNPS in ALOX12 (and so could plausibly affect the function of the gene’s protein product 12-lipooxygenase which catalyses 12-HETE synthesis); the other is rs434473 (Witola et al., 2014).

## Participants and Methods

Following approval by the University of Nigeria Teaching Hospital, Research Ethics Committee (UNTH/CSA/329/VOL.5) and informed consent/assent by the participants, 50 HbSS patients and 30 HbAA or HbAS healthy individuals who were siblings of the HbSS patients were recruited for this study. The rationale for enlisting sibling control individuals was to reduce the potential confounding effect of variations in genomic loci other than that of the beta globin (HBB) gene between the patient and control groups.

### Sample Size Calculation

The sample size was determined using the following formula (Araoye, 2004).

N = Z^2^PQ/D^2^, where N = minimum sample size, Z = Standard normal deviation set at 1.96 (for 95% confidence interval), *p* = Prevalence of homozygous sickle cell disease = 0.031. (Omotade et al., 1998).

D = Desired level of precision/standard error (0.05). Q = Point prevalence (1−*p*).

The calculated minimum sample size (N) was 46. However, the number was rounded up to 50 to make provision for possible dropouts, as observed in previous studies. (Dave et al., 2019).

### Eligibility Criteria

For both SCD patients and healthy control participants, the common inclusion criterion was age 2–60 years. The exclusion criteria for SCD patients were acute inflammatory conditions such as pneumonia, chronic inflammatory disorders such as systemic lupus erythematosus and asthma, pregnancy, breast feeding, blood transfusion in the previous 4 months, blood hemoglobin F proportion >5%, treatment with hydroxycarbamide (hydroxyurea), omega-3 fatty acids, anti-inflammatory agents or any medication that affects severity of SCD.

Medical records of HbSS patients registered in University of Nigeria Teaching Hospital were reviewed to determine the number of vaso-occlusive complications developed to date, and the number of vaso-occlusive crisis that required hospital treatment in the previous 1 year. These two disease parameters were added to obtain the vaso-occlusive events score (VOE Score). For example, in a participant who had 4 vaso-occlusive crises during the previous 1 year and, to date, has developed two vaso-occlusive complications (stroke and avascular necrosis of the head of the left femur) the VOE score is 4 + 2 = 6. To enhance the sensitivity of our method in detecting any relationship between VOE score and plasma concentrations of inflammatory molecules or ALOX12 SNPs, we recruited two clearly distinct groups of HbSS patients (without overlap in the values of SCD severity parameters used in this study) (Adegoke and Kuti, 2012; el-Hazmi, 1992; Diop et al., 1999): patients with VOE scores of 0–1 (mild disease), and those with VOE scores of three or more (severe disease). Using non-randomized consecutive sampling, 30 patients with severe SCD, 20 with mild disease and 30 HbAA or HbAS sibling control persons were recruited. The vaso-occlusive events encountered in this study were vaso-occlusive crises (painful episodes) and the following vaso-occlusive complications of SCD: cerebrovascular accident (stroke), avascular necrosis of the femoral head, sickle nephropathy including renal papillary necrosis, acute chest syndrome and priapism. The hemoglobin genotypes of the SCD patients and sibling control persons were determined by high performance liquid chromatography (HPLC), and their demographic data collected.

### Sample Collection and Laboratory Analyses

The blood sample from each SCD patient was taken during steady state with a minimum period of 30 days after the last acute illness. Venous blood (10 ml) was collected by sterile procedure into two bottles containing tri-sodium ethylenediaminetetraacetic acid (EDTA) anticoagulant. Plasma was immediately separated by centrifugation from the blood in one bottle (5 mls) and stored in 1-ml aliquots at −70°C till batched measurement of the concentrations of 12-HETE, TNF-α, and IL-1β by Enzyme-linked Immunosorbent Assay (ELISA) according to instructions from the manufacturers of the test kits [(12-HETE, Abcam ELISA kit, 133034 Lot No GR268316) (TNF-α, Abcam ELISA kit, 46087 Lot No GR262314), (IL-1β, Abcam ELISA kit, 184,861, Lot No GR260070)]**.** The second EDTA bottle of blood (5 mls) was used for automated full blood count with the Mythic 22 analyser, (Orphee, Geneva, Switzerland).

After the full blood count, the remaining blood in the second EDTA bottle was used to extract DNA from the leukocytes using Qiagen Allprep DNA/RNA Mini Kit according to the manufacturer’s instructions. The concentration of the extracted DNA was measured using NanoDrop and Qubit fluorometer to ensure sample quality. The target DNA fragment containing ALOX12 gene was amplified by Polymerase Chain Reaction (PCR) using the following primers.

For the SNP rs2073438 Forward: 5^/^TGA​GAC​CCA​AAG​AGC​AGG​TT 3^/^

Reverse: 3^/^CAA​GTC​CTC​TGC​AAC​GTC​AT 5^/^

For the SNP rs1126667 Forward: 5^/^AGT​TCC​TCA​ATG​GTG​CCA​AC 3^/^

Reverse: 3^/^CTG​CAG​CCT​TCC​TCT​GAC​TC 5^/^

### Data Analyses

A DNA sequencer software was used to analyse the data from ALOX12 gene polymorphism studies. Statistical analyses of data from the entire study were done with the IBM statistical package for social sciences (SPSS) software version 25. Frequencies, percentages, means, standard deviations and medians were used to summarize categorical and continuous variables. Associations between categorical variables were evaluated using the chi square test. Median comparison of skewed variables that do not have normal (Gaussian) distribution was done using Mann-Whitney (U) test. Pearson correlation analysis was used to test for relationship between two continuous variables. The statistical significance was set at *p* < 0.05.

## Results

### Demographics and Full Blood Count

The 50 HbSS participants included 27 (54%) males and 23 (46%) females of age 4–52 years. The 30 control individuals comprised 14 males (46.7%) and 16 females (53.3%) with mean age ±SD of 24.5 ± 11, and age range 5–57 years. There were no significant differences in age and gender distributions between the patient and control groups. The 30 patients with severe SCD included 16 males (53.3%) and 14 females (46.7%) with mean age ±SD of 24 ± 10, and age range 6–52 years. The 20 patients with mild SCD included 11 males (55%) and nine females (45%), with mean age ±SD of 18.7 ± 8.6 and age range 4–28 years. The full blood count results of the patient and control groups are summarized in Table 1. Whereas the Hb level, total WBC and platelet counts (absolute values) differed significantly between the SCD and control groups, the proportion of each leucocyte subtype (percentage as shown in Table 1) was comparable in the two groups.

**TABLE 1:** Full blood count in sickle cell disease patients and control individuals.

	Mild SCD mean ± SD	Severe SCD mean ± SD	Controls mean ± SD	*p*-value (SCD vs Controls)
Hb g/dl	8.4 ± 0.48	8.2 ± 1.19	13.4 ± 0.90	<0.001
Total WBC × 10^9^/L	9.0	11.5	4.3	<0.001
Platelet × 10^9^/L	302.0	282.7	215.4	<0.001
Neutrophil (%)	55.8 ± 3.9	58.2 ± 12.8	55.1 ± 4.5	0.343
Lymphocyte (%)	42.8 ± 2.98	39.9 ± 12.4	45.3 ± 6.8	0.067
Monocyte (%)	0.85 ± 1.23	0.90 ± 1.40	1.37 ± 1.22	0.267
Eosinophil (%)	0.45 ± 0.69	0.73 ± 1.05	1.13 ± 1.33	0.092
Basophil (%)	0.05 ± 0.22	0.17 ± 0.53	0.22 ± 0.61	0.581

### Variation of Plasma Concentration of Pro-Inflammatory Molecules with Severity of SCD

**FIGURE 1:**
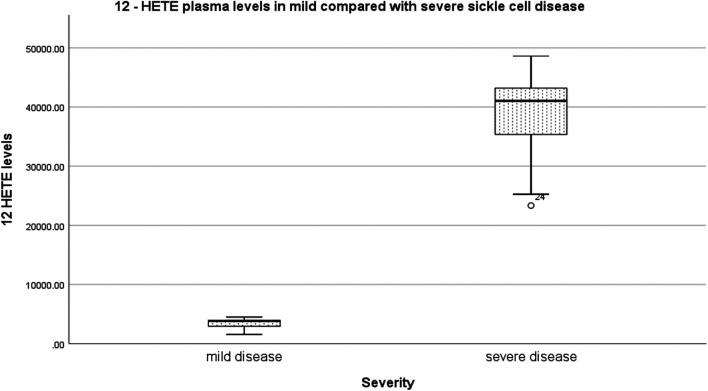
Variation of plasma concentration (pg/ml) of 12-HETE with SCD severity. The thick horizontal line in each box represents the median 12-HETE value for the disease severity group.

**FIGURE 2:**
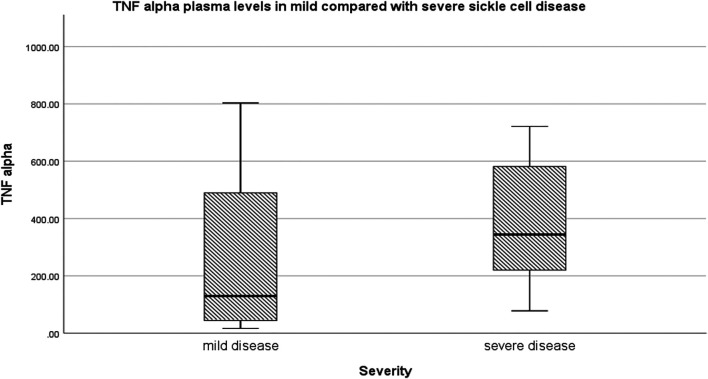
Variation of plasma concentration (pg/ml) of TNF-alpha with SCD severity. The thick horizontal line in each box represents the median value of TNF-α for the disease severity group.

**FIGURE 3:**
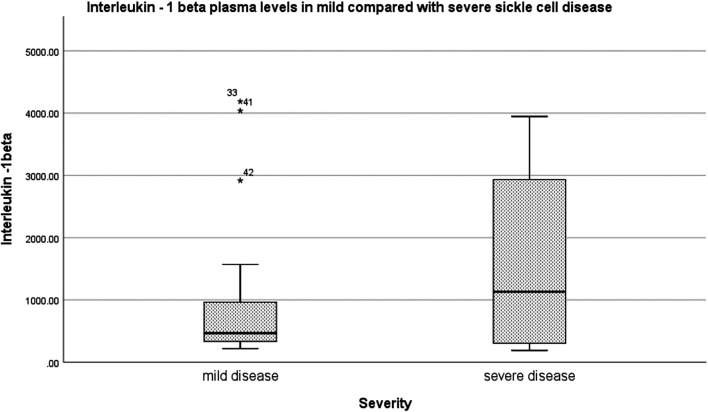
Variation of plasma concentration (pg/ml) of IL-1β with SCD severity. The thick horizontal line in each box represents the median value of IL-1β for the disease severity group.

Relative to mild SCD patients (VOE score 0–1), those with severe disease (VOE score ≥3) had higher plasma levels of 12-HETE (*p* < 0.0001) and TNF-α (*p* = 0.019), but not IL-1β (*p* = 0.277); Table 2. The effect size is large for 12-HETE (0.84), moderate for TNF-α (0.32), and small for IL-1β (0.15). Variation of plasma concentration of the pro-inflammatory molecules with SCD severity is illustrated in Figures 1–3. In addition, compared to their sibling control group, the cohort of all SCD patients had significantly high plasma levels of 12-HETE (*p* < 0.0001), TNF-α (*p* < 0.0001), and IL-1β (*p* < 0.0001); two-tailed Mann-Whitney *U* test.

**TABLE 2:** Plasma levels (pg/ml) of pro-inflammatory molecules in severe *versus* mild SCD.

	Median plasma Concentration			
	Mild SCD	Severe SCD	Control group	*p*-value (mild vs severe SCD) (Mann-Whitney *U* test, 2-tailed)
12- HETE	3,797.50	41,041.64	1,658.50	<0.0001
TNF alpha	129.93	344.31	45.44	0.019
IL-1β	466.38	1,131.78	270.41	0.277

### Correlation of VOE Score with Plasma Concentration of 12-HETE, TNF-alpha and IL-1β

There was a strong and highly significant direct correlation between vaso-occlusive events score and plasma concentration of the pro-inflammatory molecule 12-HETE (r = 0.65, *p* < 0.0001), Table 3. The correlation between VOE score and TNF-α or IL-1β was not statistically significant.

**TABLE 3:** Correlation of VOE Score with plasma levels of pro-inflammatory molecules.

Statistics	12-HETE	TNF-alpha	IL-1beta
Spearman Correlation Coefficient	0.65	0.26	0.06
95% Confidence Interval	0.45–0.79	−0.02–0.51	−0.22–0.34
*p*-value	<0.0001	0.06	0.66
N	50	50	50

**FIGURE 4:**
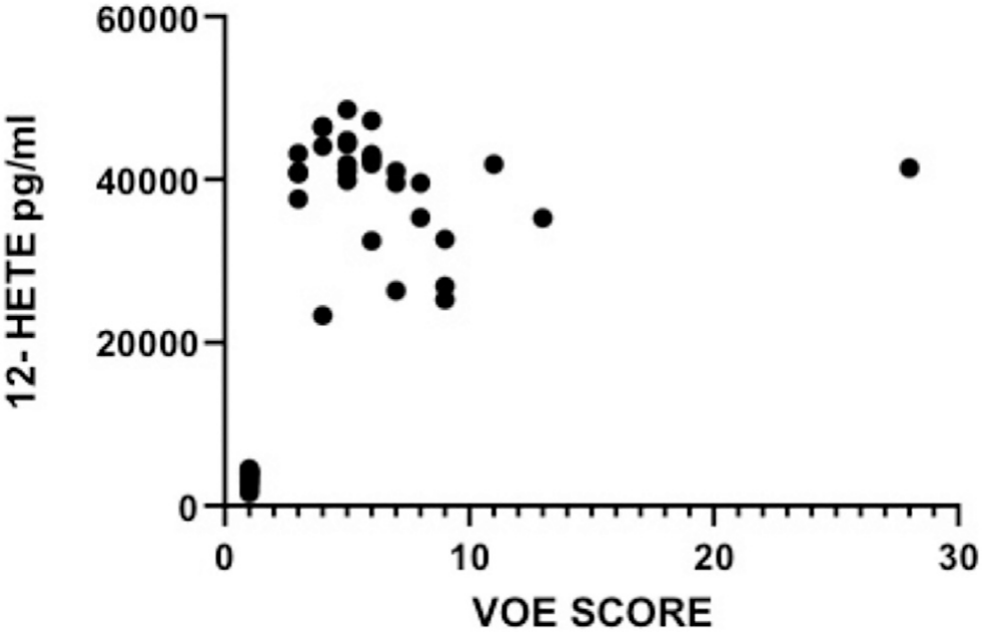
Plasma 12-HETE levels in relation to VOE Scores of sickle cell disease patients.

The plasma levels of 12-HETE in relation with VOE scores are depicted in Figure 4. The VOE scores and plasma concentrations of the three pro-inflammatory molecules in all SCD patients are shown in Table 4.

**TABLE 4:** Vaso-occlusive events scores and plasma cytokine levels (pg/ml) in sickle cell disease.

Study ID number	VOE score	12-HETE	TNF-α	IL-1β
1	4	44,086.55	513.87	2,616.85
2	4	46,398.08	721.6	453.46
3	5	44,762.55	597.9	632.08
4	5	41,887.95	588.56	274.84
5	5	41,041.64	289.81	277.79
6	7	26,381.15	196.45	660.87
7	28	41,436.25	170.77	188.48
8	3	37,602.45	584.59	2,912.83
9	9	25,254.45	551.22	1762.86
10	13	35,290.15	341.16	299.94
11	6	43,071.5	161.44	3,368.24
12	11	41,887.95	327.85	246.79
13	5	44,311.85	434.75	263.03
14	9	32,696.75	170.77	3,309.93
15	7	39,575.65	212.79	305.1
16	6	42,564	397.17	1,628.53
17	7	41,041.64	572.22	330.2
18	5	48,597.35	252.46	3,532.84
19	6	47,243.35	347.46	2,933.5
20	3	40,928.875	77.65	285.17
21	6	32,471.35	621.24	3,385.22
22	3	43,184.15	310.81	539.08
23	3	40,703.35	180.11	3,946.18
24	4	23,337.2	628.24	2,594.71
25	8	39,575.665	219.79	1,602.69
26	6	41,943.775	579.93	3,606.65
27	4	46,510.85	327.85	3,563.1
28	8	35,346.9	593.23	477.08
29	5	39,857.585	243.13	314.7
30	9	26,945.75	581.56	2,559.28
31	1	3,827.5	250.13	435.01
32	1	2,728.5	133.43	497.75
33	1	3,831.1	742.61	4,186.06
34	1	2,789.3	341.16	522.84
35	1	2,126.57	803.29	1,161.3
36	1	2,908.5	126.43	764.94
37	1	3,902.2	761.28	300.67
38	1	3,365.4	726.27	389.25
39	1	3,767.5	173.11	522.11
40	1	2,993.8	637.58	365.63
41	1	1,555.5	45.2	4,036.23
42	1	4,149.7	16.5	2,917.26
43	1	3,168.5	81.85	685.23
44	1	4,104.4	167.04	1,570.22
45	1	3,839.1	44.27	243.84
46	1	3,827.8	123.16	393.67
47	1	4,256.5	16.5	286.65
48	1	3,974.1	44.27	375.22
49	1	3,382.4	16.96	246.79
50	1	4,515.6	29.8	218.01

### Single Nucleotide Polymorphisms of ALOX12 Gene in Relation to Severity of SCD

**FIGURE 5:**
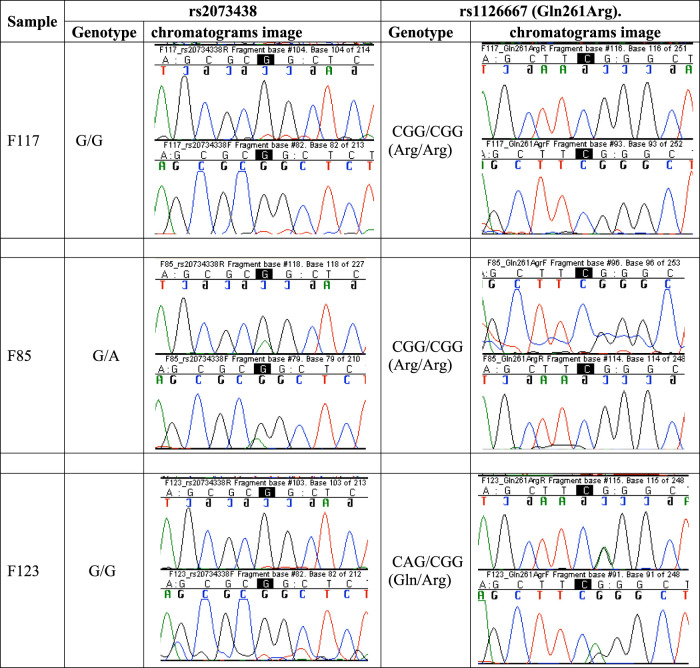
Chromatogram images showing results of DNA sequencing to determine the genotypes at SNPs rs2073438 and rs1126667 of ALOX12 gene. Participant F117 is homozygous G/G at rs2073438 and homozygous CGG/CGG at rs1126667, F85 is heterozygous G/A at rs2073438, and F123 heterozygous CAG/CGG at rs1126667. DNA sequencing results for all participants are accessible at https://www.frontiersin.org/articles/10.3389/fgeed.2021.722190/full#supplementary-material.

Figure 5 is a set of chromatogram images showing the results of sequencing the DNA samples of three study participants to illustrate how the genotypes at the ALOX12 SNPs rs2073438 and rs1126667 were determined.

#### SNP rs2073438

In the group of 30 patients with severe SCD (VOE score ≥3), 29 (96.7%) were homozygous G/G at the rs2073438 position; and 1 (3.3%) was heterozygous G/A. Among the 20 patients with mild SCD (VOE score 0–1), 19 (95%) were homozygous G/G; and 1 (5%) heterozygous G/A. All the HbAA and HbAS siblings of the SCD patients were homozygous G/G at the rs2073438 position. The proportions of homozygous (G/G) and heterozygous (G/A) individuals with mild or severe SCD were not significantly different (χ^2^ = 1.37, *p* = 0.5); Table 5.

**TABLE 5:** ALOX12 SNP genotypes in relation to the severity of sickle cell disease.

SNP/Genotype	Mild SCD n (%)	severe SCD	sibling Control group	χ^2^	*p* value
rs2073438					
G/G	19 (95.0)	29 (96.7)	30 (100.0)	1.37	0.51
G/A	1 (5.0)	1 (3.3)	0 (0.0)		
Total	20 (100)	30 (100)	30 (100)		
rs1126667					
CGG/CGG	8 (40.0)	17 (56.7)	14 (46.7)	1.42	0.49
CAG/CGG	12 (60.0)	13 (43.3)	16 (53.3)		
Total	20 (100)	30 (100)	30 (100)		

When all homozygous G/G patients are put in one group, and all heterozygous G/A patients in another group, comparison of the two (homozygous G/G vs heterozygous G/A) groups did not show any significant difference in the proportions of patients with mild or severe SCD (χ^2^ = 0.087, *p* = 0.77). Therefore, no significant relationship was found between the severity of SCD and the genotype (G/G or G/A) at the rs2073438 SNP.

#### SNP rs1126667

In the severe SCD group, 17/30 (56.7%) were homozygous CGG/CGG; 13 (43.3%) had the heterozygous genotype CAG/CGG. In the mild SCD group, 8/20 (40%) were homozygous CGG/CGG, and 12 (60%) heterozygous CAG/CGG. Among their 30 sibling controls, 14 (46.7%) were homozygous CGG/CGG; and 16 (53.3%) heterozygous CAG/CGG. The proportions of homozygous (CGG/CGG) and heterozygous (CAG/CGG) individuals were not significantly different in the mild or severe SCD groups (χ^2^ = 1.42, *p* = 0.49), Table 4. When all homozygous CGG/CGG patients are put in one group, and all heterozygous CAG/CGG patients in another group, none of the two groups showed any significant difference in the proportions of patients with mild *versus* severe SCD (χ^2^ = 1.333, *p* = 0.248). So, neither the homozygous CGG/CGG genotype of SNP rs1126667, nor the heterozygous CAG/CGG genotype, was found to be significantly associated with either mild or severe SCD.

### Comparison of VOE Scores in SCD Patients with Different Genotypes of ALOX12 SNPs

Severe SCD patients who were homozygous G/G at rs2073438 had a median vaso-occlusive event score of 6, and the heterozygous G/A ones a median score of 5. The VOE scores in homozygous patients with severe SCD were not significantly different from those of the heterozygotes who had severe disease (*U* = 10.5, *p* = 0.64). With respect to SNP rs1126667, homozygous (CGG/CGG) persons with severe SCD had a median VOE score of 6; and the heterozygous (CAG/CGG) individuals a median score of 5. The VOE scores in the two groups were not significantly different (*U* = 96.5, *p* = 0.55). All patients with mild SCD had VOE scores of 1, and so there was no difference in VOE score between homozygous compared with heterozygous mild SCD patients with respect to the two ALOX12 SNPs evaluated in this study.

### Plasma Concentration of 12-HETE in Relation to Genotypes of SNPs in ALOX12 Gene

#### rs2073438

Homozygous G/G genotype was found in 78/80 (97.5%) of all participants in this study, and the heterozygous G/A genotype in only 2/80 (2.5%). No participant had A/A genotype. The number of heterozygous participants was too small for statistical analysis.

#### rs1126667

By contrast, the genotypes of SNP rs1126667 were more evenly distributed among the participants, with homozygous CGG/CGG of 49% (39/80) and heterozygous CAG/CGG of 51% (41/80). Plasma 12-HETE concentrations in homozygous and heterozygous SCD patients were not significantly different (Table 6). Similarly, there was no significant difference in plasma 12-HETE levels of all 39 homozygous participants (SCD patients and sibling control persons, median = 3,831 pg/ml), and all 41 heterozygous subjects (median 3,169 pg/ml) in this study (Mann-Whitney *U* = 679, *p* = 0.248, 2-tailed).

**TABLE 6:** Plasma 12-HETE levels in SCD patients with different genotypes of rs1126667.

	Homozygous CGG/CGG	Heterozygous CAG/CGG	Statistical tests results		*p*—value
	41,887.95	35,346.9	Mann-Whitney U	236.00	0.140 (Exact, 2-tailed)
	46,398.08	41,887.95	Wilcoxon W	561.00	
	44,762.55	25,254.45	Z	−1.48	
	32,696.75	42,564.1			
	44,086.55	46,510.85			
	41,041.64	47,243.35			
	41,436.25	40,928.87			
	37,602.45	26,381.15			
	32,290.15	23,337.2			
	44,311.85	39,575.66			
	41,943.77	32,471.35			
	43,184.15	39,857.58			
	26,945.75	41,041.64			
	40,703.35	2,728.5			
	48,597.35	4,104.4			
	43,071.5	4,256.5			
	39,575.65	3,382.4			
	1,555.5	3,168.5			
	3,902.2	2,908.5			
	3,831.1	2,789.3			
	2,126.57	4,515.6			
	3,827.5	3,767.5			
	3,365.4	2,993.8			
	3,827.8	4,149.7			
	3,974.1	3,839.1			
N	25	25			
Median	39,576 pg/ml	23,337 pg/ml			

## Discussion

The very significantly higher plasma concentration of 12-hydroxyeicosatetraenoic acid in severe relative to mild SCD, and the markedly strong direct correlation of vaso-occlusive events score with the plasma level of this pro-inflammatory molecule suggest that its clinical relevance in SCD-associated vaso-occlusion deserves to be evaluated. It is considered that 12-HETE is elevated in SCD as part of the inflammatory response to recurrent tissue ischemia and/or infarction caused by vaso-occlusion in various parts of the body. This concept is in keeping with the raised levels of various inflammatory markers, during the steady state of SCD. The clinical relevance of inflammation as a predisposing factor to vaso-occlusive events in SCD is underlined by clinical experience and observations from numerous research studies that infection (which causes inflammation) is the most common precipitating factor for vaso-occlusive crisis (painful episodes) in affected individuals. (Buchanan and Glader, 1978; Samir et al., 1996; Okpala, 2004b). As a multi-system disease that has at least three fundamental mechanisms (vaso-occlusion, hemolysis and immune compromise) acting separately or in synergy to cause illness, SCD has the prospect of being ameliorated by inhibiting the generalized and continual obstruction of blood vessels that causes ischemic damage/infarction and dysfunction in several organs of the body. A pro-inflammatory product of arachidonic acid metabolism the biosynthesis of which is catalyzed by the enzyme 12-Lipooxygenase encoded by ALOX12 gene, 12-HETE was not previously recognized as a strong correlate of the number of vaso-occlusive events in SCD. Previous investigations by Setty and co-workers had shown that plasma 12-HETE is raised in steady-state SCD and rises higher during vaso-occlusive crisis (Setty et al., 1998), whereas the currently reported study provided the distinct new information that steady-state (or baseline) plasma level of this pro-inflammatory molecule directly correlates with the number of vaso-occlusive events in this inherited condition.

That plasma levels of 12-HETE and TNF-α levels were significantly increased in severe compared to mild SCD supports the current school of thought that inflammation is an important component of the pathophysiology of this haemoglobinopathy. From this perspective, SCD could be considered as a chronic inflammatory condition and therapy designed to inhibit inflammation could confer clinical benefit to affected persons. Increased blood levels of pro-inflammatory and anti-inflammatory molecules have been observed in several previous studies of SCD in the relatively less symptomatic steady state, and further elevated during episodes of acute illness such as vaso-occlusive crisis. (Francis and Haywood, 1992; Hedo et al., 1993; Carlos and Harlan, 1994; Springer, 1994; Samir et al., 1996; Setty et al., 1998; Belcher et al., 2000; Platt, 2000; Osarogiagbon, 2001; Okpala, 2006; Micheal et al., 2009; Conran and Belcher, 2018; Piccin et al., 2019; Sundd et al., 2019; Nader et al., 2020). Elevated plasma levels of 12-HETE have been found in other conditions of chronic inflammation and oxidative stress. (Sarah et al., 2013; Shan et al., 2015). Cytokines, such as TNF-α, have a role in the activation of leucocytes, especially monocytes and neutrophils, in SCD. Activation of leucocytes and release of cytokines stimulate the nuclear factor kappa β transcription factor pathway, which controls the synthesis of both anti-inflammatory (e.g, IL-4) and pro-inflammatory (e.g, IL-6, IL-8) cytokines. (Tian et al., 2005; Thassila Nogueira et al., 2013; Sameh et al., 2015). The significantly increased plasma level of IL-1β in SCD patients relative to their sibling controls found in this study is consistent with the findings from previous work by Musa et al. (Musa et al., 2010) The cytokines TNF-α and IL-1β impair blood flow and impede recovery from ischaemic episodes by increasing adhesion of sickled erythrocytes to endothelium; (Tian et al., 2005); and so cytokine imbalance is implicated in the pathogenesis of sickle cell crisis. Alteration of cytokine balance (increased pro-inflammatory and reduced anti-inflammatory molecules) occurs in SCD. (Musa et al., 2010; Thassila Nogueira et al., 2013; Sameh et al., 2015). During vaso-occlusive crisis, there is increase in plasma levels of the pro-inflammatory cytokines IL-1β, TNF-α, IL-6, IL-8, IL-15, IL-16, IL-17, and IL-18. Anti-inflammatory cytokines with raised plasma levels in steady-state SCD patients compared to those in vaso-occlusive crisis, or to healthy individuals, include IL-4, IL-10, IL-11 and IL-13. It is noteworthy that the difference in age range between mild SCD patients in the current study (4–28 years) and those with severe disease (6–52 years) could affect the findings on cytokine levels because the concentration of plasma proteins could vary with age, and such variation might confound the observations from this study and their interpretation.

The two ALOX12 SNPS (rs2073438 and rs1126667) evaluated in this study were chosen because the former has been associated with raised 12-HETE levels in urine, and the latter is one of the only two common missense SNPS with a potential to affect the function of the enzyme protein product of the gene (the other is rs434473) (Witola et al., 2014). No relationship was found between either SNP and the number of vaso-occlusive events or plasma 12-HETE level in this study of SCD patients. However, other ALOX12 SNPs had shown association with disease states in previous studies (Witola et al., 2014). In the light of this, more variants of the ALOX12 gene will be evaluated in subsequent studies. Also, neither rs2073438 which was associated with raised urinary12-HETE in a previous investigation (Kaul et al., 1996), nor the missense rs126667, showed any relationship with plasma concentration of this pro-inflammatory molecule in the current study. The reason for the variance between the findings of the previous and current studies is not clear.

Consistent with previous reports, leucocyte and platelet counts were significantly higher in the SCD patients compared with their sibling control participants in this study. Leucocytes and platelets contribute to inflammation and the formation of heterocellular aggregates that cause vaso-occlusion in SCD (Kaul et al., 1989; Hedo et al., 1993; Carlos and Harlan, 1994; Springer, 1994; Kaul et al., 1996; Franklin, 1997; Belcher et al., 2000; Platt, 2000; Osarogiagbon, 2001; Okpala, 2004b; Frenette, 2004; Okpala, 2006; Conran and Belcher, 2018; Piccin et al., 2019; Sundd et al., 2019; Nader et al., 2020). High neutrophil count is associated with severe sickle cell disease. (Anyaegbu et al., 1998). From the perspective of haemodynamics, high leucocyte and platelet counts (Okpala, 2002), and raised plasma concentrations of acute phase plasma proteins (such as fibrinogen) that occur during inflammation, all would increase the blood viscosity, reduce the velocity of blood flow in the microvasculature, and predispose to vaso-occlusion.

## Conclusion

Taken together, the results of this study indicate that inflammation contributes to the development of vaso-occlusion in SCD. The clinical importance of the pro-inflammatory molecule 12-HETE, the plasma level of which was found to be a strong correlate of the number of vaso-occlusive events in SCD, deserves to be evaluated in further studies.

## Data Availability

The original contributions presented in the study are included in the article/Supplementary Material, further inquiries can be directed to the corresponding author.
