# Supplementary material for: Plasma Concentration of 12-Hydroxyeicosatetraenoic Acid, Single Nucleotide Polymorphisms of 12-Lipooxygenase Gene and Vaso-Occlusion in Sickle Cell Disease
Source: Front Genome Ed. 2021 Aug 26;3:722190. doi: 10.3389/fgeed.2021.722190 (PMC8525407; doi:10.3389/fgeed.2021.722190)
Supplement: Supplementary file 1 [file DataSheet1.ZIP › ALOX12 ORF and primers.docx]

**Primers for Gln261Arg of ALOX12**

OLIGO [start](http://flypush.imgen.bcm.tmc.edu/primer/primer3_www_results_help.html#PRIMER_START)  [len](http://flypush.imgen.bcm.tmc.edu/primer/primer3_www_results_help.html#PRIMER_LEN)  [tm](http://flypush.imgen.bcm.tmc.edu/primer/primer3_www_results_help.html#PRIMER_TM)  [gc%](http://flypush.imgen.bcm.tmc.edu/primer/primer3_www_results_help.html#PRIMER_GC)  [any](http://flypush.imgen.bcm.tmc.edu/primer/primer3_www_results_help.html#PRIMER_ANY)  [3'](http://flypush.imgen.bcm.tmc.edu/primer/primer3_www_results_help.html#PRIMER_REPEAT) [seq](http://flypush.imgen.bcm.tmc.edu/primer/primer3_www_results_help.html#PRIMER_OLIGO_SEQ)

LEFT PRIMER 47 20 59.97 50.00 4.00 3.00 AGTTCCTCAATGGTGCCAAC

RIGHT PRIMER 257 20 60.28 60.00 6.00 3.00 ctgcagccttcctctgactc

SEQUENCE SIZE: 310

INCLUDED REGION SIZE: 310

PRODUCT SIZE: 211, PAIR ANY COMPL: 4.00, PAIR 3' COMPL: 0.00

1 gAGAAGGTTCGCCAGTGCTGGCAGGATGATGAGTTGTTCAGCTACCAGTTCCTCAATGGT

>>>>>>>>>>>>>>

61 GCCAACCCCATGCTGTTGAGACGCTCGACCTCTCTGCCCTCCAGGCTAGTGCTGCCCTCG

>>>>>>

121 GGGATGGAAGAGCTT**CAG**GCTCAACTGGAGAAAGAACTTCAGgtacctctccttcccctg

181 cctggcactgttctctccttagtagtgtggtgagaacggacagagagaatccaaactgag

<<<

241 tcagaggaaggctgcagtactgcattgccccttggtgcagtcctgtgctggaaaaataca

<<<<<<<<<<<<<<<<<

301 gaaataaaat

**** Green: primers; red: mutation; blue: amplicon**

**Primers for rs2073438 of ALOX12**

OLIGO [start](http://flypush.imgen.bcm.tmc.edu/primer/primer3_www_results_help.html#PRIMER_START)  [len](http://flypush.imgen.bcm.tmc.edu/primer/primer3_www_results_help.html#PRIMER_LEN)  [tm](http://flypush.imgen.bcm.tmc.edu/primer/primer3_www_results_help.html#PRIMER_TM)  [gc%](http://flypush.imgen.bcm.tmc.edu/primer/primer3_www_results_help.html#PRIMER_GC)  [any](http://flypush.imgen.bcm.tmc.edu/primer/primer3_www_results_help.html#PRIMER_ANY)  [3'](http://flypush.imgen.bcm.tmc.edu/primer/primer3_www_results_help.html#PRIMER_REPEAT) [seq](http://flypush.imgen.bcm.tmc.edu/primer/primer3_www_results_help.html#PRIMER_OLIGO_SEQ)

LEFT PRIMER 55 20 59.84 50.00 3.00 0.00 tgagacccaaagagcaggtt

RIGHT PRIMER 247 20 58.88 50.00 4.00 2.00 CAAGTCCTCTGCAACGTCAT

SEQUENCE SIZE: 300

INCLUDED REGION SIZE: 300

PRODUCT SIZE: 193, PAIR ANY COMPL: 4.00, PAIR 3' COMPL: 2.00

1 cagcatcagctccaggcgctgcgggccctcgtcctccacccgactccgggcgtctgagac

>>>>>>

61 ccaaagagcaggttgtgcgggggcgggaacgaggcgatgctgtctttggaggccctgaga

>>>>>>>>>>>>>>

121 aactgaggttgcacaggagcgc**g**gctctgtcctcgaaacggcctcagtcgggtccctcct

181 actaagtctggcctgggtccggcctgcacagGAGGAGGAGTTTGATCATGACGTTGCAGA

<<<<<<<<<<<<<

241 GGACTTGGGGCTCCTGCAGTTCGTGAGGCTGCGCAAGCACCACTGGCTGGTGGACGACGC

<<<<<<<

**** Green: primers; red: mutation; blue: amplicon**

**ALOX12 ORF**

Translation Position: 1 - 1992;

Genetic Code : Universal

10 20 30 40 50 60

ATGGGCCGCTACCGCATCCGCGTGGCCACCGGGGCCTGGCTCTTCTCCGGGTCGTACAAC

M G R Y R I R V A T G A W L F S G S Y N

70 80 90 100 110 120

CGCGTGCAGCTTTGGCTGGTCGGGACGCGCGGGGAGGCGGAGCTGGAGCTGCAGCTGCGG

R V Q L W L V G T R G E A E L E L Q L R

130 140 150 160 170 180

CCCGCGCGGGGCGA**GG**AGGAGGAGTTTGATCATGACGTTGCAGAGGACTTGGGGCTCCTG

P A R G E E E E F D H D V A E D L G L L

190 200 210 220 230 240

CAGTTCGTGAGGCTGCGCAAGCACCACTGGCTGGTGGACGACGCGTGGTTCTGCGACCGC

Q F V R L R K H H W L V D D A W F C D R

250 260 270 280 290 300

ATCACGGTGCAGGGCCCTGGAGCCTGCGCGGAGGTGGCCTTCCCGTGCTACCGCTGGGTG

I T V Q G P G A C A E V A F P C Y R W V

310 320 330 340 350 360

CAGGGCGAGGACATCCTGAGCCTGCCCGAGGGCACC**GC**CCGCCTGCCAGGAGACAATGCT

Q G E D I L S L P E G T A R L P G D N A

370 380 390 400 410 420

TTGGACATGTTCCAGAAGCATCGAGAGAAGGAACTGAAAGACAGACAGCAGATCTACT**GC**

L D M F Q K H R E K E L K D R Q Q I Y C

430 440 450 460 470 480

TGGGCCACCTGGAAGGAAGGGTTACCCCTGACCATCGCTGCAGACCGTAAGGATGATCTA

W A T W K E G L P L T I A A D R K D D L

490 500 510 520 530 540

CCTCCAAATATGAGATTCCATGAGGAGAAGAGGCTGGACTTTGAATGGACACTGAAGGCA

P P N M R F H E E K R L D F E W T L K A

550 560 570 580 590 600

G**GG**GCTCTGGAGATGGCCCTCAAACGTGTTTACACCCTCCTGAGCTCCTGGAACTGCCTA

G A L E M A L K R V Y T L L S S W N C L

610 620 630 640 650 660

GAAGACTTTGATCAGATCTTCTGGGGCCAGAAGAGTGCCCTGGCT**GA**GAAGGTTCGCCAG

E D F D Q I F W G Q K S A L A E K V R Q

670 680 690 700 710 720

TGCTGGCAGGATGATGAGTTGTTCAGCTACCAGTTCCTCAATGGTGCCAACCCCATGCTG

C W Q D D E L F S Y Q F L N G A N P M L

730 740 750 760 770 780

TTGAGACGCTCGACCTCTCTGCCCTCCAGGCTAGTGCTGCCCTCGGGGATGGAAGAGCTT

L R R S T S L P S R L V L P S G M E E L

790 800 810 820 830 840

CAGGCTCAACTGGAGAAAGAACTTCA**GA**ATGGTTCCCTGTTTGAAGCTGACTTCATCCTT

**Q**  A Q L E K E L Q N G S L F E A D F I L

850 860 870 880 890 900

CTGGATGGAATTCCAGCCAACGTGATCCGAGGAGAGAAGCAATACCTGGCTGCCCCCCTC

L D G I P A N V I R G E K Q Y L A A P L

910 920 930 940 950 960

GTTATGCTGAAGATGGAGCCCAATGGGAAGCTGCAGCCCATGGTCATCCAGATTCAGCCT

V M L K M E P N G K L Q P M V I Q I Q P

970 980 990 1000 1010 1020

CCCAACCCCAGCTCTCCAACCCCAACACTGTTCCTGCCCTCAGACCCCCCACTTGCCTGG

P N P S S P T P T L F L P S D P P L A W

1030 1040 1050 1060 1070 1080

CTCCTGGCAAAGTCCTGGGTCCGAAATTCAGATTTCCAACTGCACGAGATCCAGTATCAC

L L A K S W V R N S D F Q L H E I Q Y H

1090 1100 1110 1120 1130 1140

TTGCTGAACACTCACCTGGTGGCTGAGGTCATCGCTGTCGCCACCATGCGGTGCCTCCCA

L L N T H L V A E V I A V A T M R C L P

1150 1160 1170 1180 1190 1200

GGACTGCACCCCATCTTCAAGTTCCTGATCCCCCATATCCGCTACACCATGGAAATCAAC

G L H P I F K F L I P H I R Y T M E I N

1210 1220 1230 1240 1250 1260

ACCCGGGCCCGGACCCAACTCATCTCAGATGGAGGAATTTTTGATAAGGCAGTGAGCACA

T R A R T Q L I S D G G I F D K A V S T

1270 1280 1290 1300 1310 1320

GGTGGAGGGGGCCATGTACAGTTGCTCCGTCGGGCGGCAGCTCAGCTGACCTACTGCTCC

G G G G H V Q L L R R A A A Q L T Y C S

1330 1340 1350 1360 1370 1380

CTCTGTCCTCCTGACGACCTGGCTGACCGGGGCCTGCTGGGACTCCCAGGTGCTCTCTAT

L C P P D D L A D R G L L G L P G A L Y

1390 1400 1410 1420 1430 1440

GCCCATGATGCTTTACGGCTCTGGGAGATCATTGCCAGGTATGTGGAGGGGATCGTCCAC

A H D A L R L W E I I A R Y V E G I V H

1450 1460 1470 1480 1490 1500

CTCTTCTACCAAAGGGATGACATAGTGAAGGGGGACCCTGAGCTGCAGGCCTGGTGTCGG

L F Y Q R D D I V K G D P E L Q A W C R

1510 1520 1530 1540 1550 1560

GAGATCACGGAGGTGGGGCTGTGCCAGGCCCAGGACCGAGGTTTCCCTGTCTCCTTCCAG

E I T E V G L C Q A Q D R G F P V S F Q

1570 1580 1590 1600 1610 1620

TCCCAGAGTCAACTCTGCCATTTCCTCACCATGTGCGTCTTCACGTGCACTGCCCAGCAT

S Q S Q L C H F L T M C V F T C T A Q H

1630 1640 1650 1660 1670 1680

GCCGCCATCAACCAGGGCCAGCTGGACTGGTATGCCTGGGTCCCTAATGCTCCATGCACA

A A I N Q G Q L D W Y A W V P N A P C T

1690 1700 1710 1720 1730 1740

ATGCGGATGCCCCCACCCACCACCAAGGAAGATGTGACGATGGCCACAGTGATGGGGTCA

M R M P P P T T K E D V T M A T V M G S

1750 1760 1770 1780 1790 1800

CTACCTGATGTCCGGCAGGCCTGTCTTCAAATGGCCATCTCATGGCATCTGAGTCGCCGC

L P D V R Q A C L Q M A I S W H L S R R

1810 1820 1830 1840 1850 1860

CAGCCAGACATGGTGCCTCTGGGGCACCACAAAGAAAAATATTTCTCAGGCCCCAAGCCC

Q P D M V P L G H H K E K Y F S G P K P

1870 1880 1890 1900 1910 1920

AAAGCTGTGCTAAACCAATTCCGAACAGATTTGGAAAAGCTGGAAAAGGAGATTACAGCC

K A V L N Q F R T D L E K L E K E I T A

1930 1940 1950 1960 1970 1980

CGGAATGAGCAACTTGACTGGCCCTATGAATATCTGAAGCCCAGCTGCATAGAGAACAGT

R N E Q L D W P Y E Y L K P S C I E N S

1990 2000

GTCACCATCTGA

V T I *
