# Supplementary material for: Plasma Concentration of 12-Hydroxyeicosatetraenoic Acid, Single Nucleotide Polymorphisms of 12-Lipooxygenase Gene and Vaso-Occlusion in Sickle Cell Disease
Source: Front Genome Ed. 2021 Aug 26;3:722190. doi: 10.3389/fgeed.2021.722190 (PMC8525407; doi:10.3389/fgeed.2021.722190)
Supplement: Supplementary file 1 [file DataSheet1.ZIP › City of Hope 1.pdf]

CITY OF HOPE NATIONAL MEDICAL CENTER

Molecular Pathology Core Laboratory (TRL)
Department of Pathology
Raju K. Pillai, MD, Director

Sample	rs2073438		Gln261Arg	
	**genotype**	chromatograms image	genotype	chromatograms image
F117	G	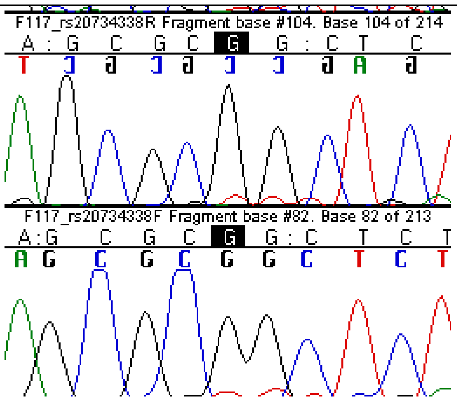	CGG	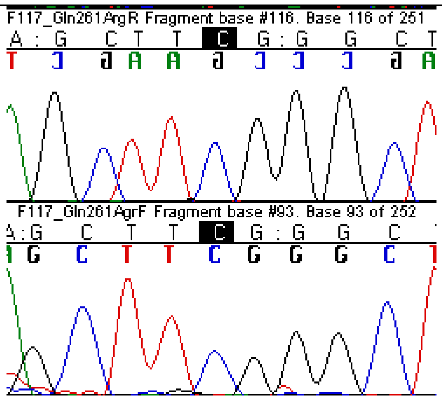
F85	G/A	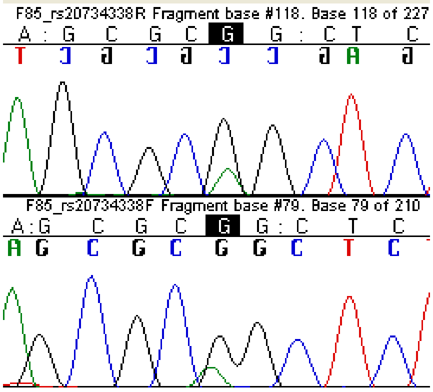	CGG	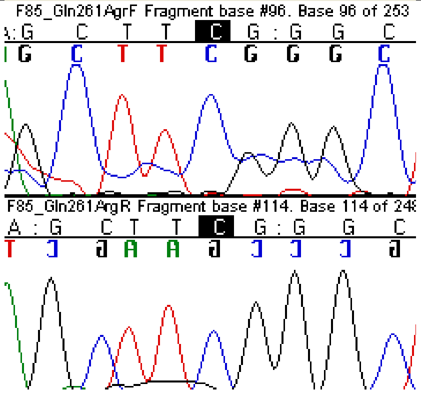
F102	G	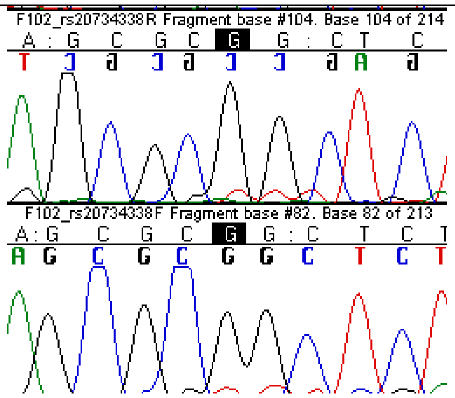	CGG	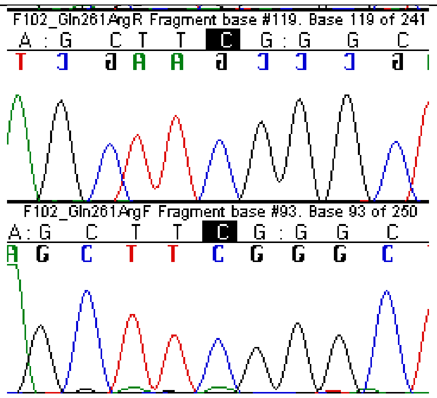

F143	G	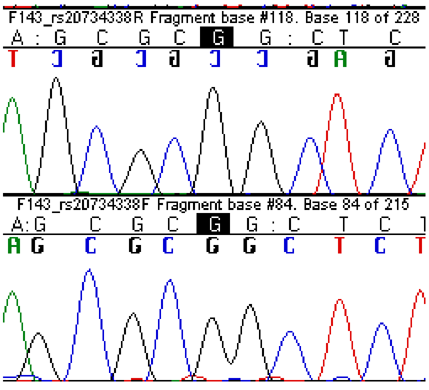	CGG	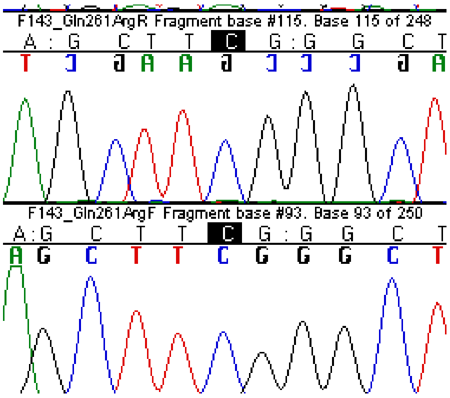
F136	G	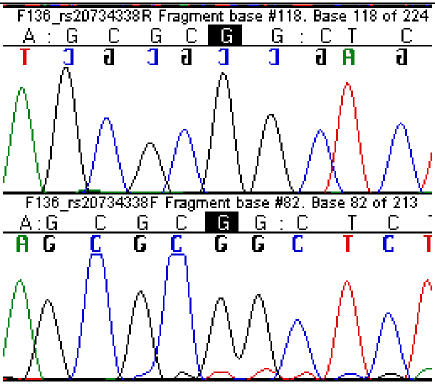	CAG/CGG	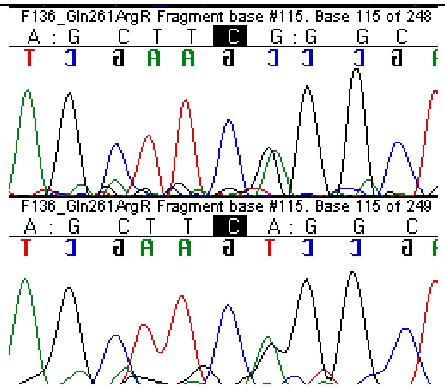
F137	G	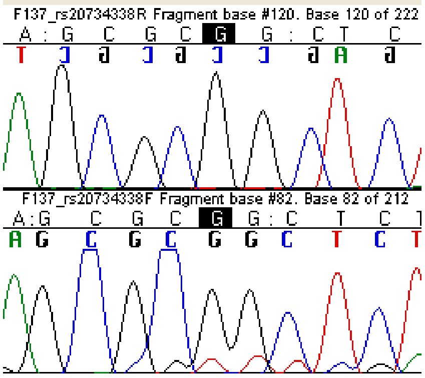	CAG/CGG	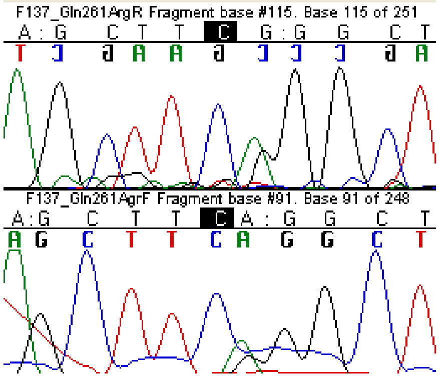
F123	G	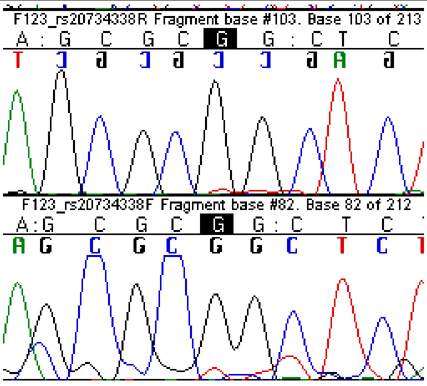	CAG/CGG	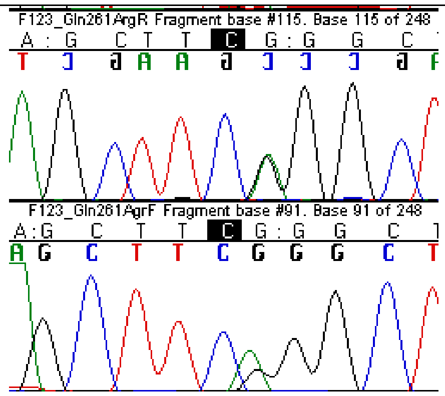

F125	G	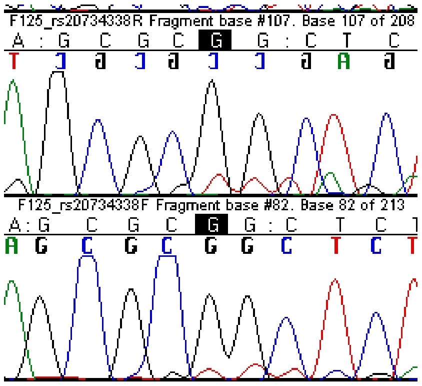	CAG/CGG	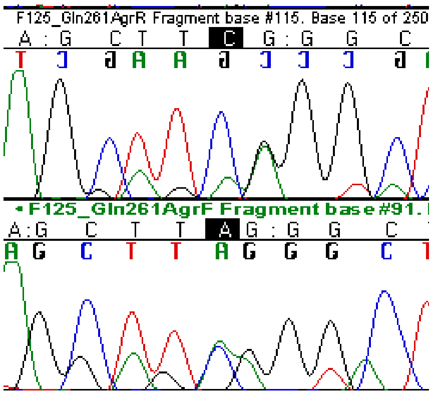
F135	G	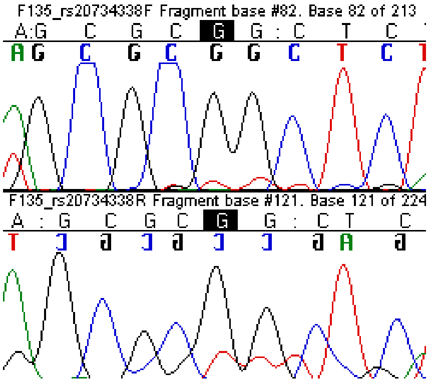	CAG	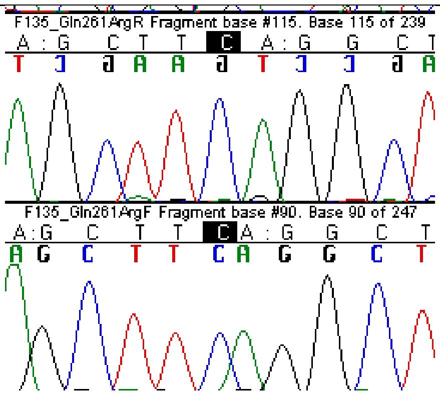
F119	G	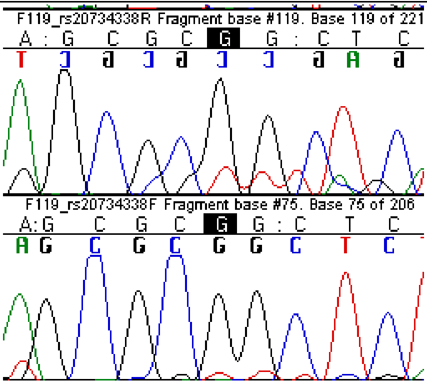	CAG/CGG	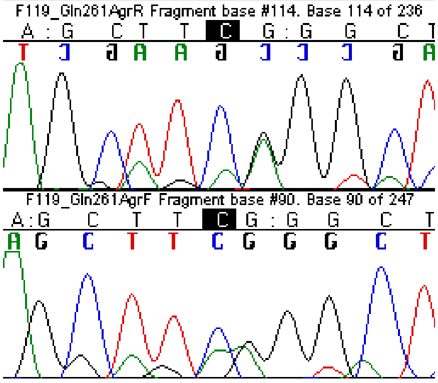
F140	G	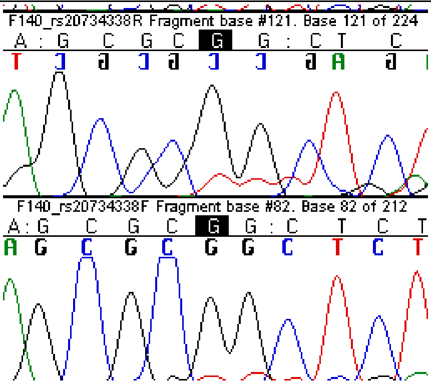	CAG/CGG	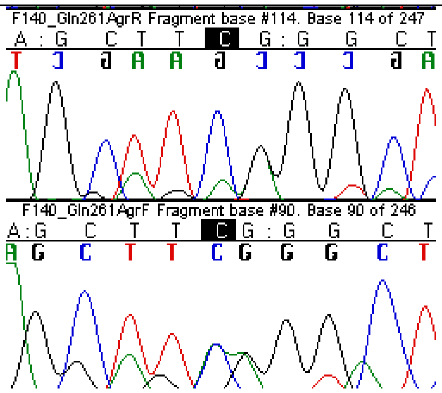

F86	G	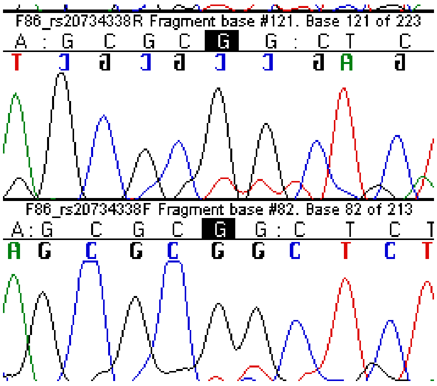	CGG	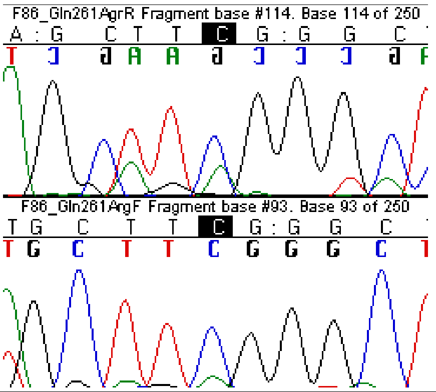
F144	G	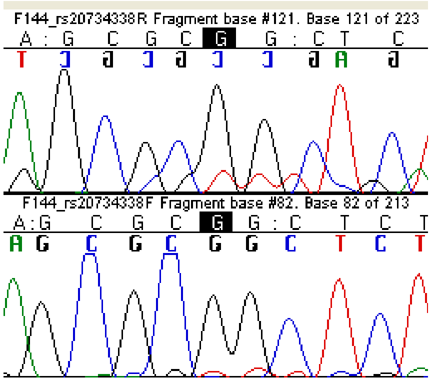	CAG/CGG	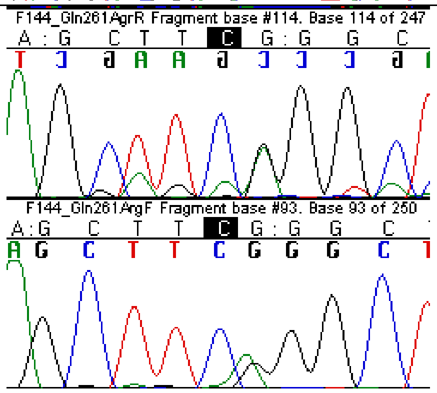
F90	G	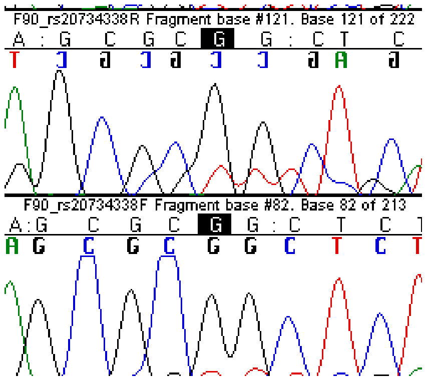	CGG	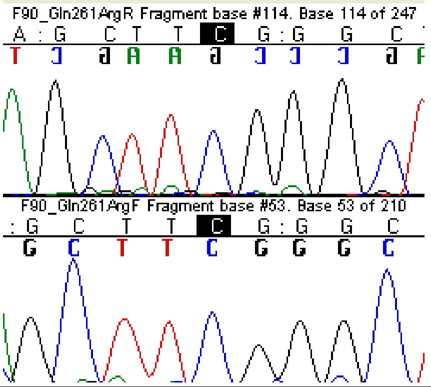
F103	G	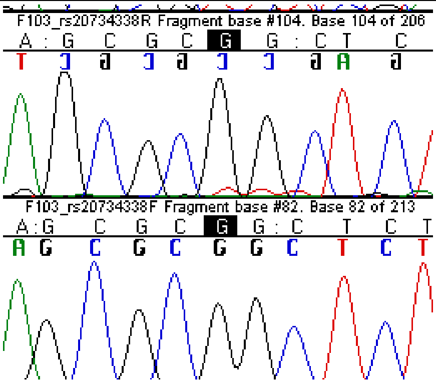	CAG/CGG	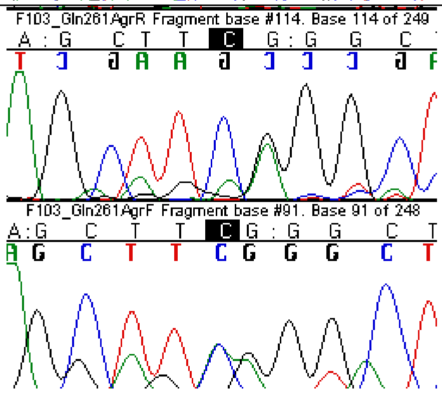

F21	G	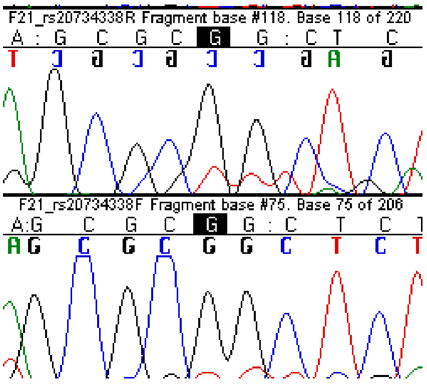	CGG	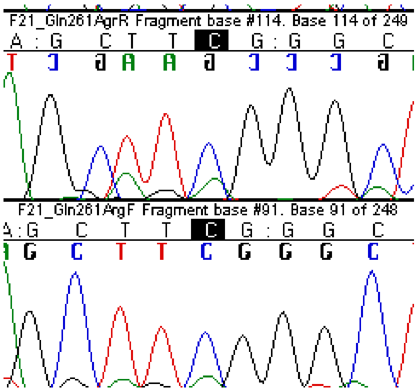
P164	G	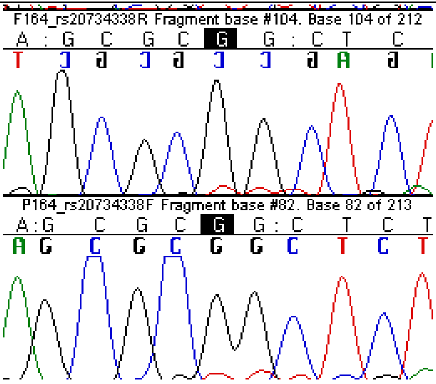	CGG	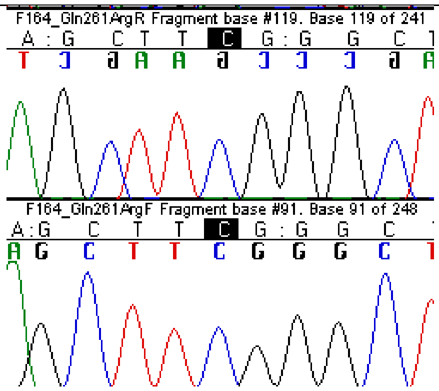
DA1	G	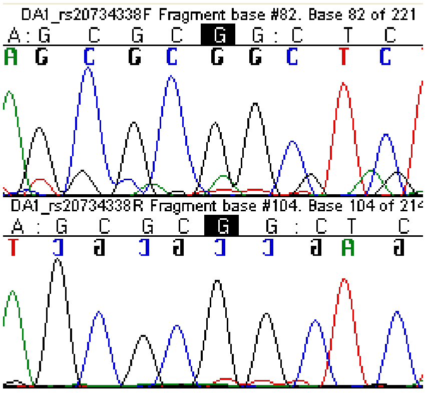	CAG/CGG	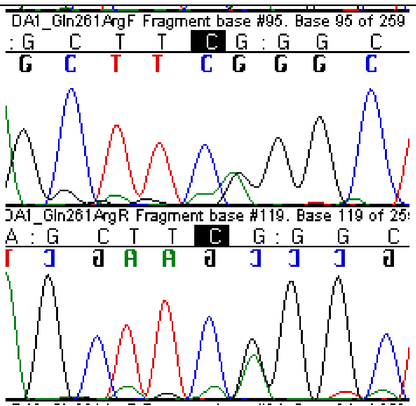
DA2	G	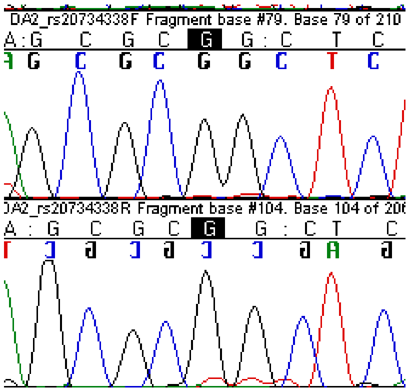	CAG/CGG	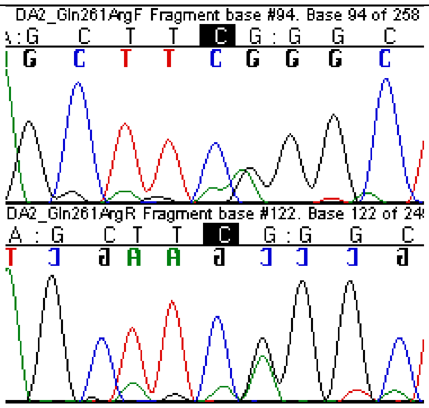

DA3	G	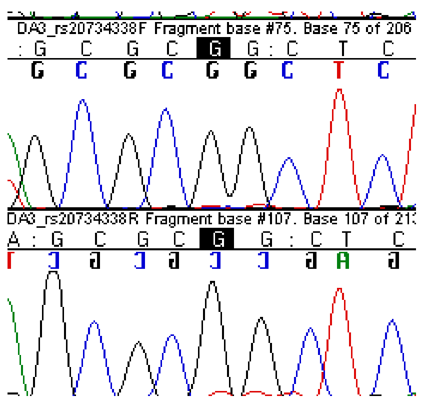	CGG	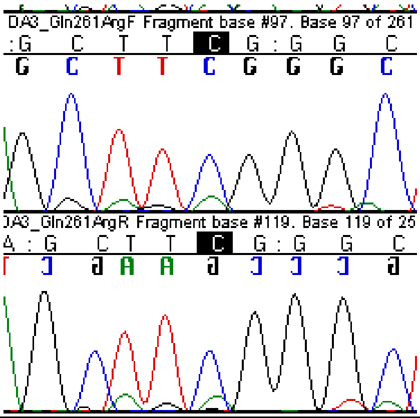
DA4	G	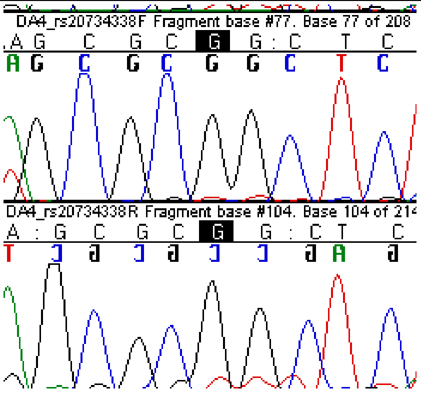	CAG/CGG	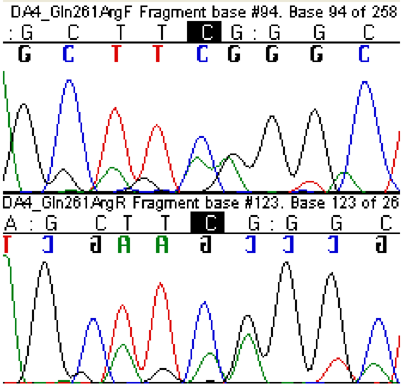
DA5	G	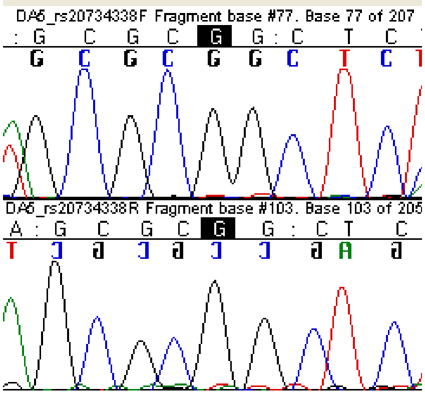	CGG	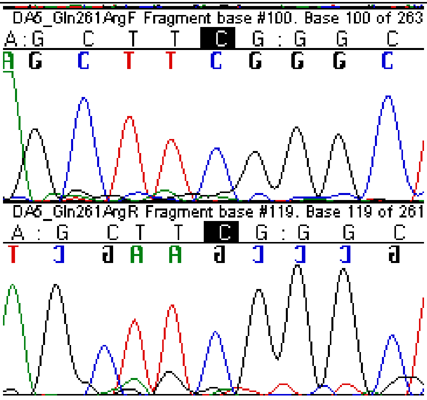
DA6	G	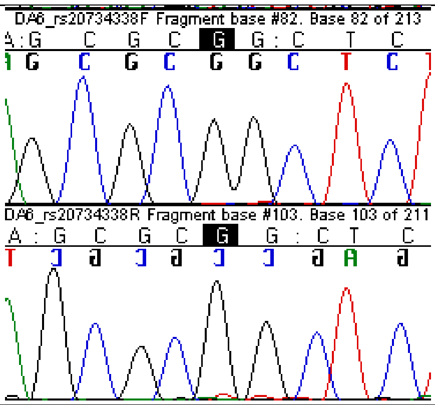	CGG	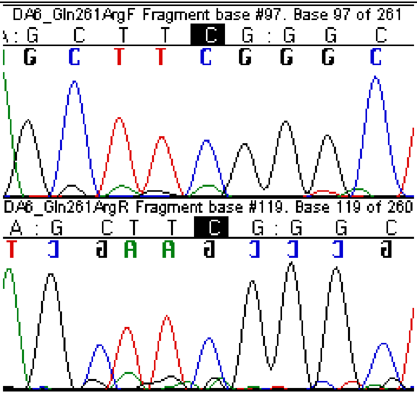

DA7	G	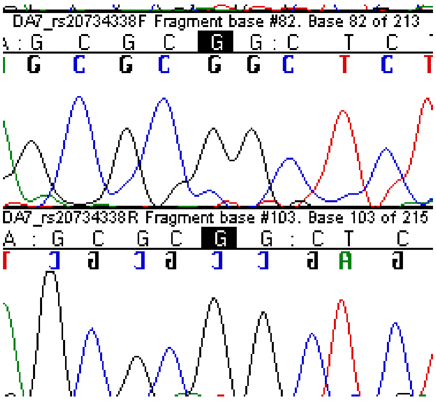	CAG/CGG	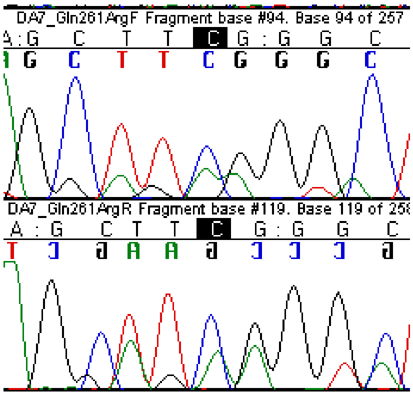
DA8	G	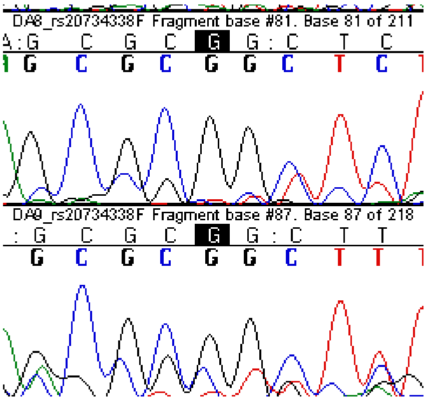	CAG/CGG	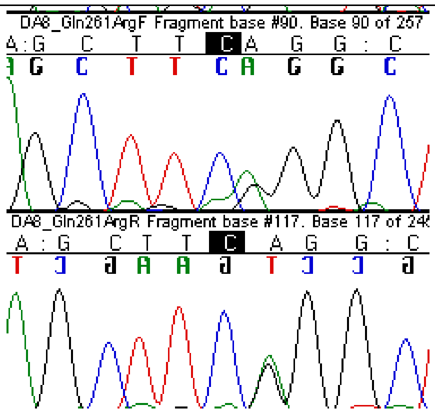
DA9	G	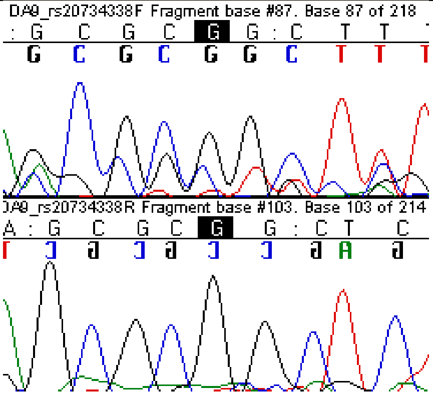	CGG	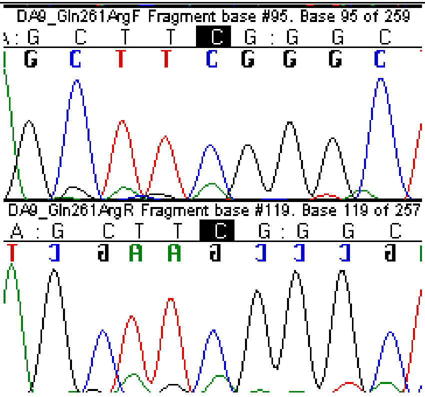
DA10	G	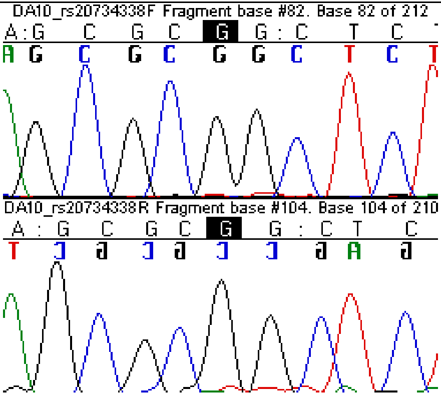	CGG	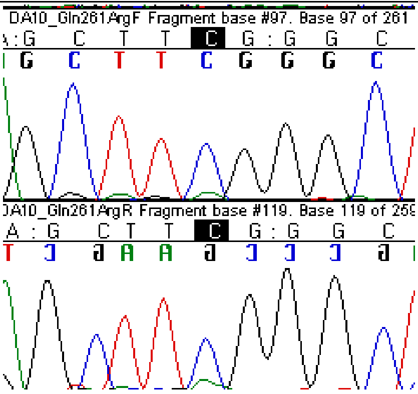

DA11	G	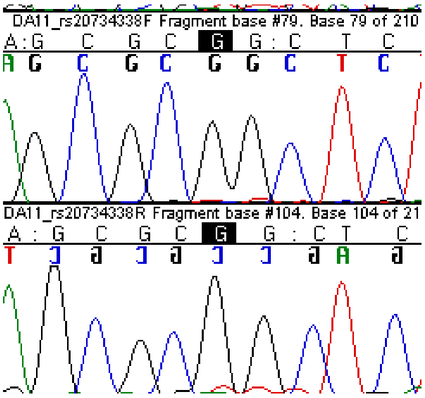	CGG	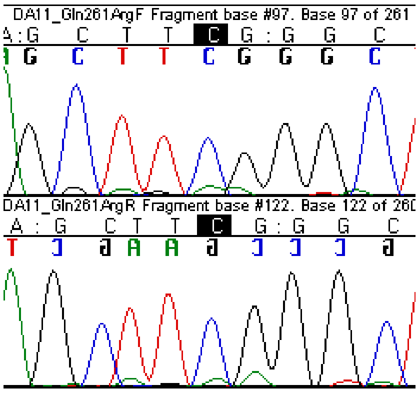
DA12	G	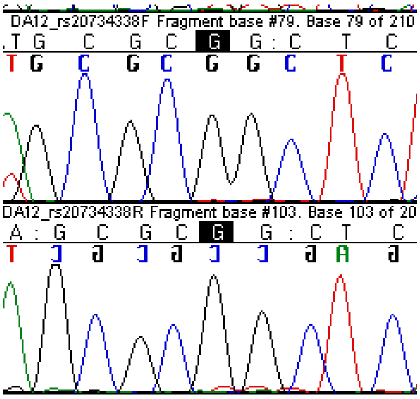	CAG/CGG	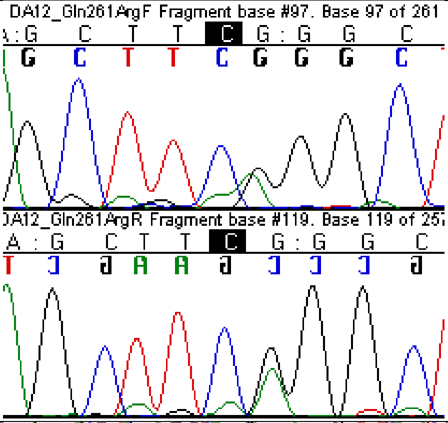
DA13	G	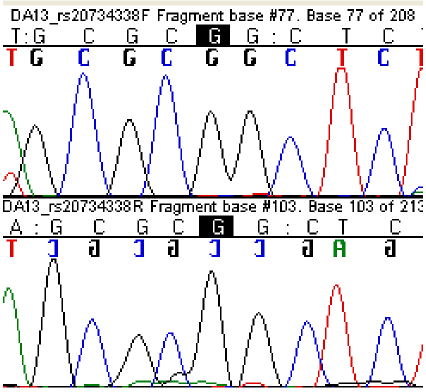	CGG	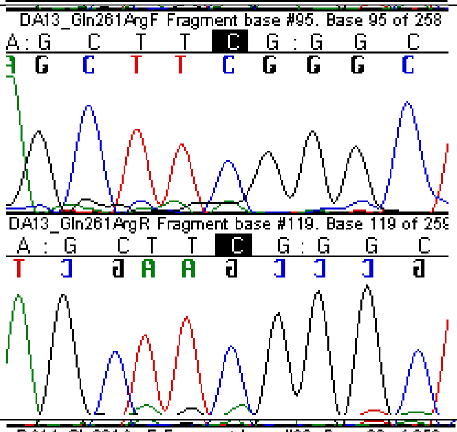
DA14	G	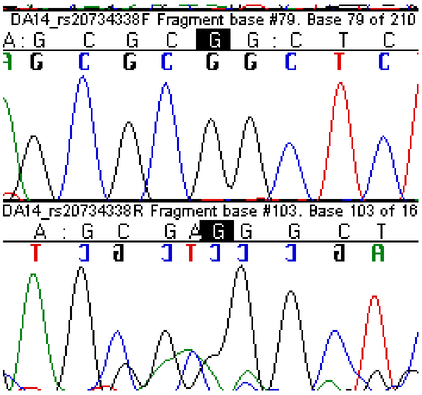	CAG/CGG	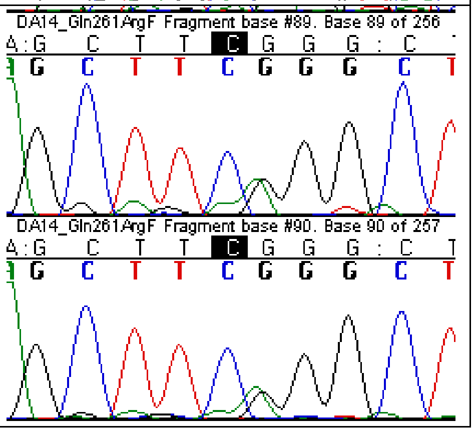

DA15	G	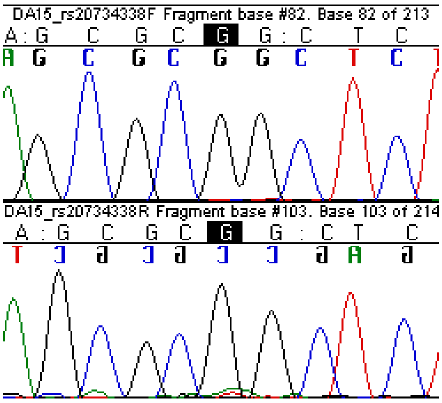	CAG/CGG	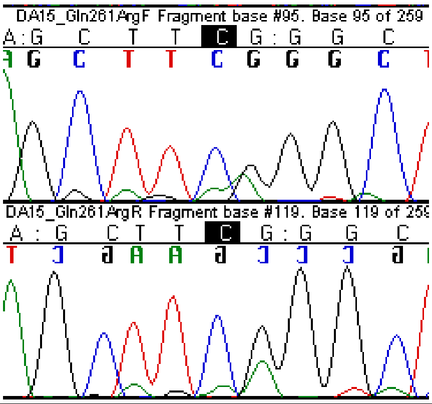
DA16	G	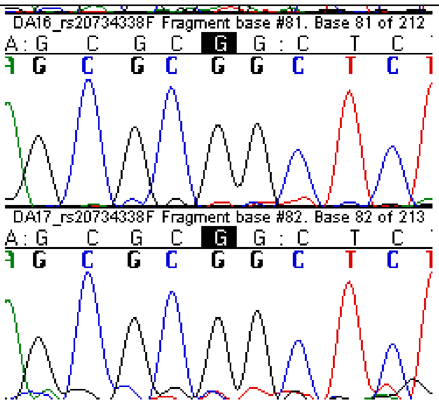	CGG	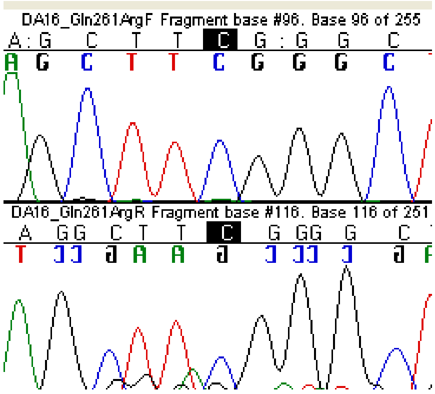
DA17	G	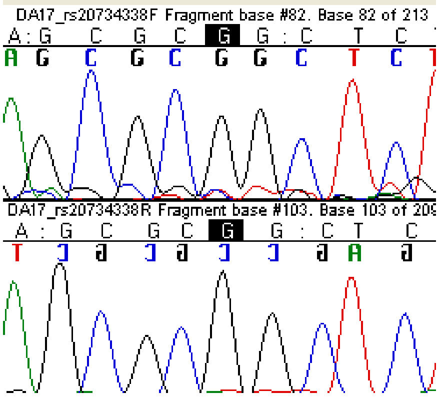	CAG/CGG	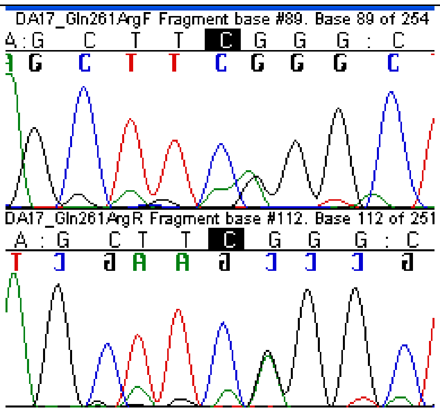
DA18	G	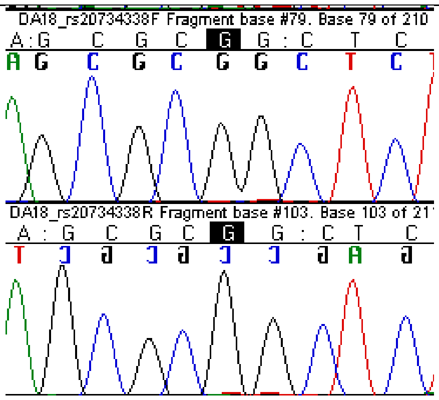	CGG	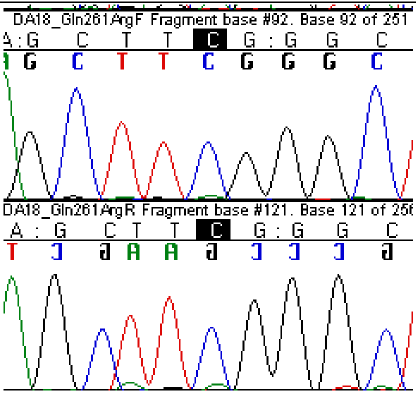

DA19	G	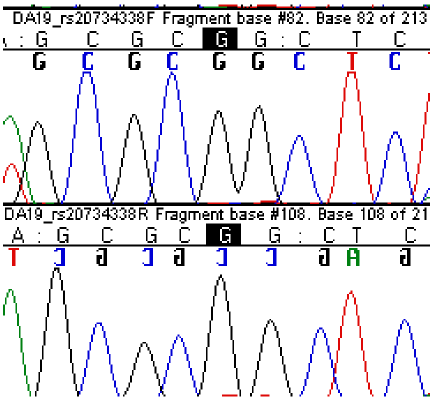	CGG	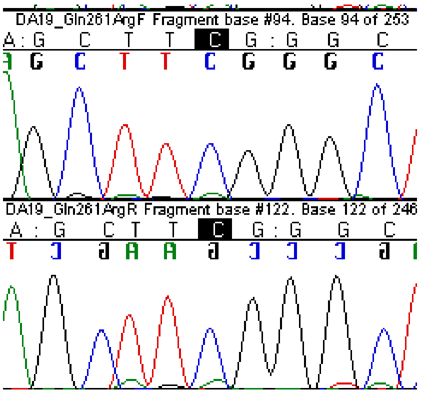
DA20	G	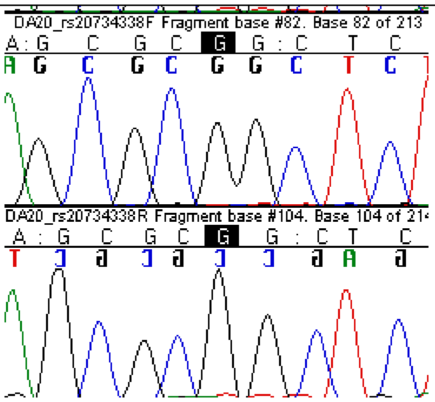	CAG/CGG	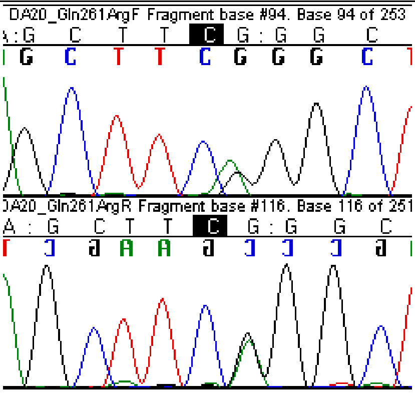
DA21	G	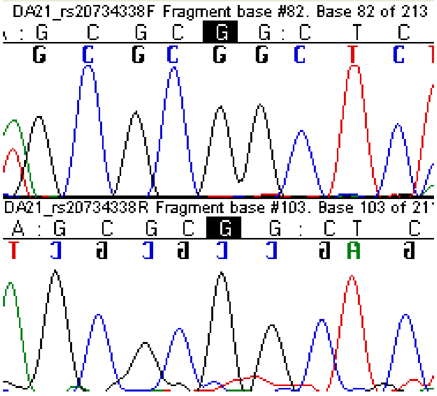	CAG/CGG	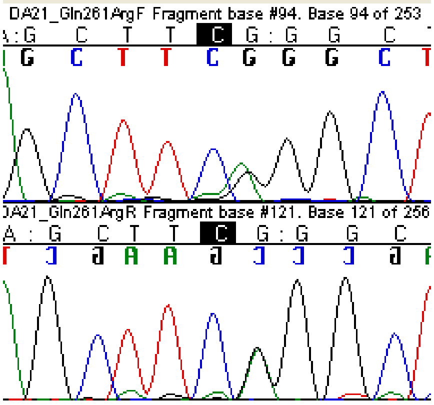
DA22	G	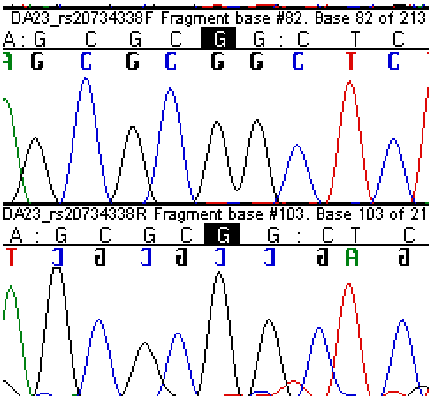	CAG/CGG	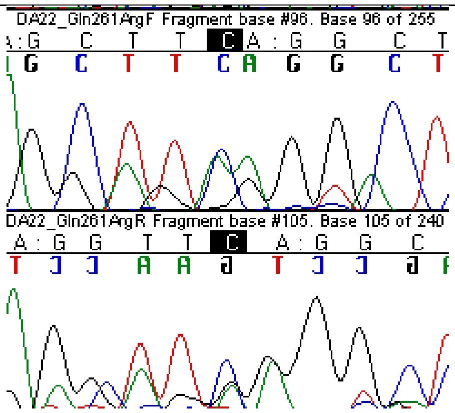

DA23	G	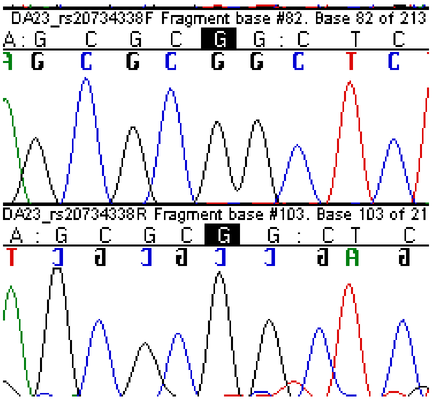DA23_rs20734338F Fragment base #82. Base 82 of 213<br>DA23_rs20734338R Fragment base #103. Base 103 of 21	CGG	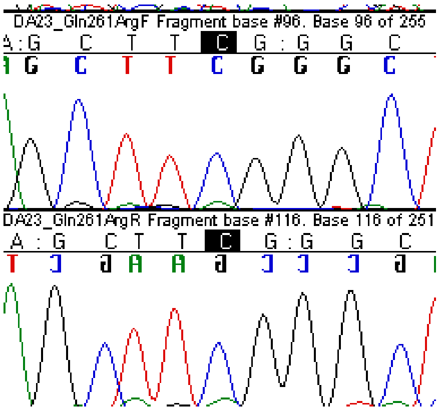DA23_Gln261ArgF Fragment base #96. Base 96 of 255<br>DA23_Gln261ArgR Fragment base #116. Base 116 of 251
