# Supplementary material for: Plasma Concentration of 12-Hydroxyeicosatetraenoic Acid, Single Nucleotide Polymorphisms of 12-Lipooxygenase Gene and Vaso-Occlusion in Sickle Cell Disease
Source: Front Genome Ed. 2021 Aug 26;3:722190. doi: 10.3389/fgeed.2021.722190 (PMC8525407; doi:10.3389/fgeed.2021.722190)
Supplement: Supplementary file 1 [file DataSheet1.ZIP › City of Hope 2.pdf]

Sample	**rs2073438**		Gln261Arg	
	**genotype**	chromatograms image	genotype	chromatograms image
F111	G	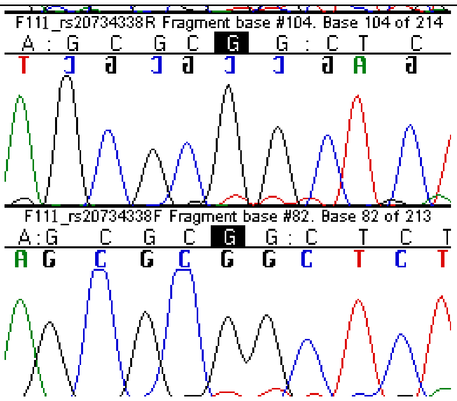	CGG	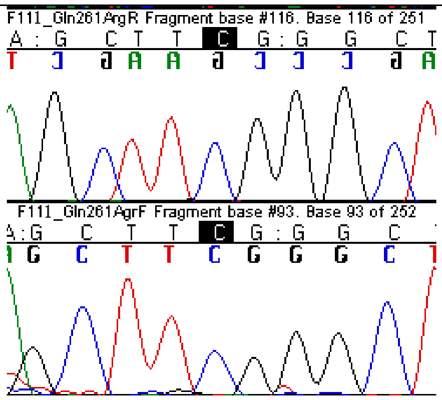
F113	G/A	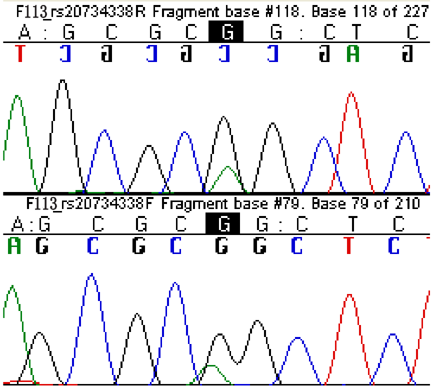	CGG	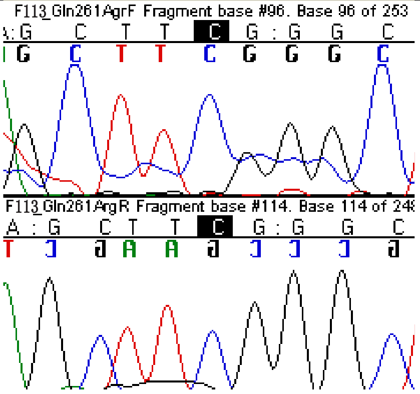
F112	G	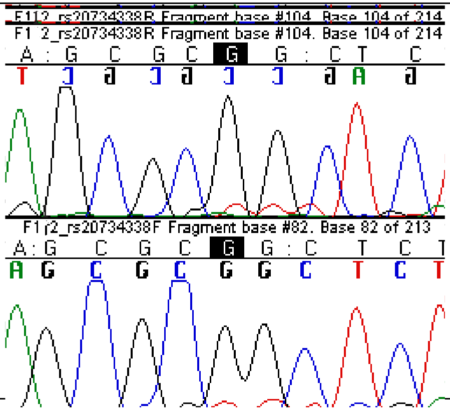	CGG	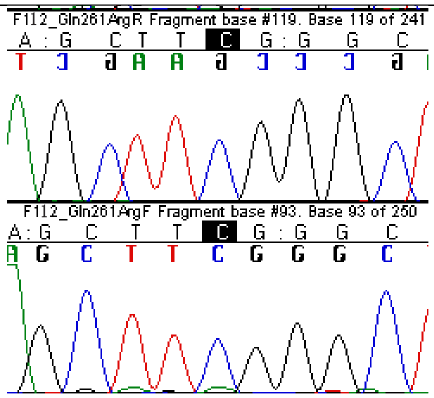

F105	G	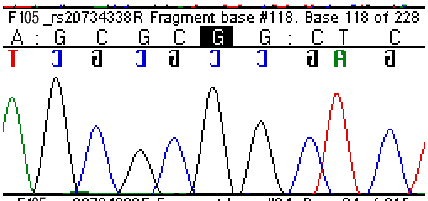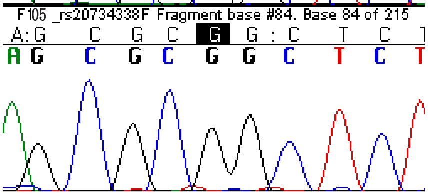	CGG	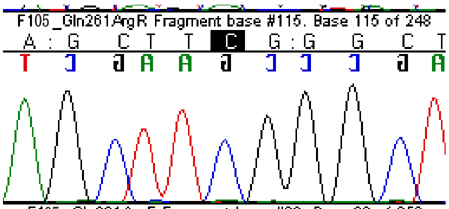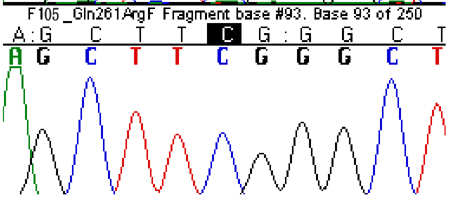
F146	G	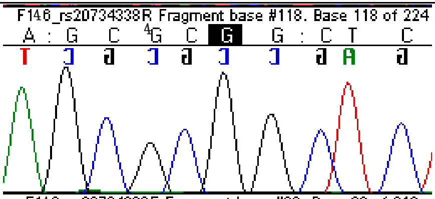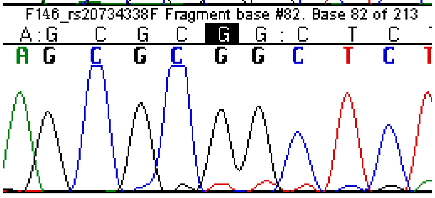	CAG/CGG	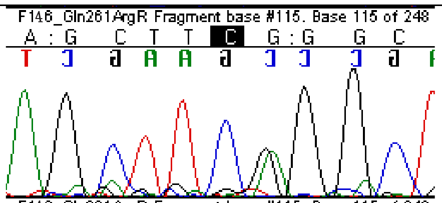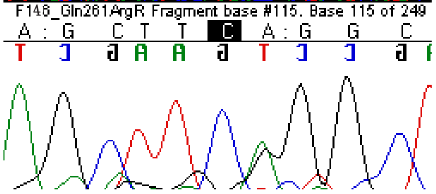
F108	G	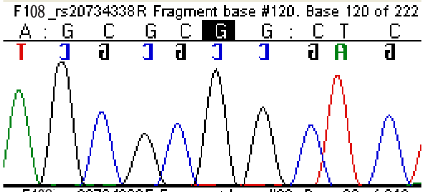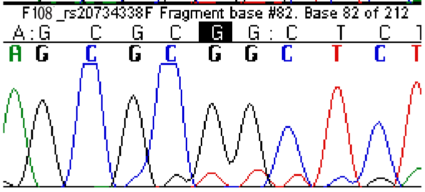	CAG/CGG	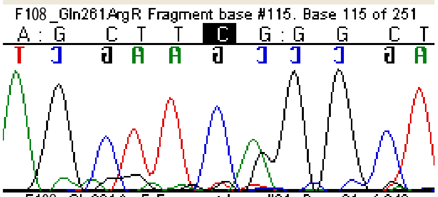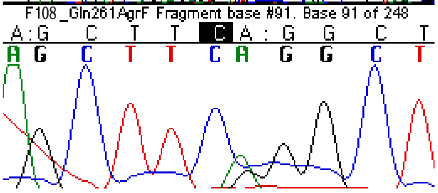
F100	G	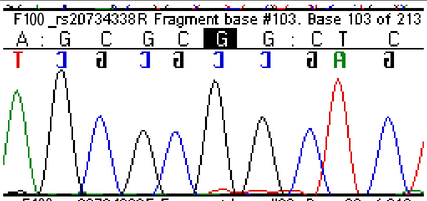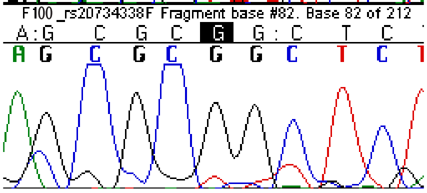	CAG/CGG	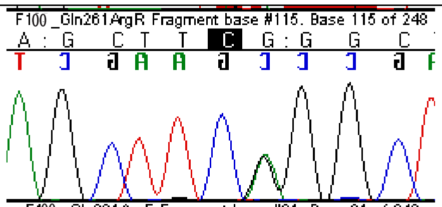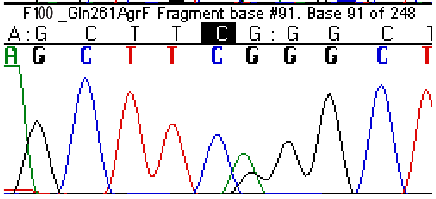

F115	G	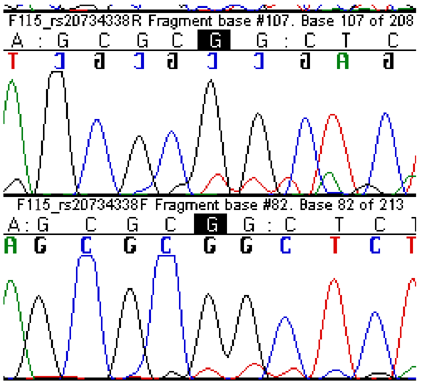	CAG/CGG	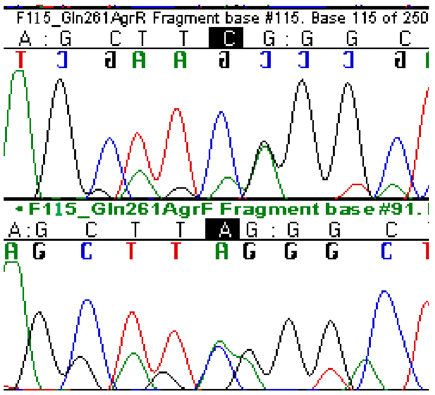
F141	G	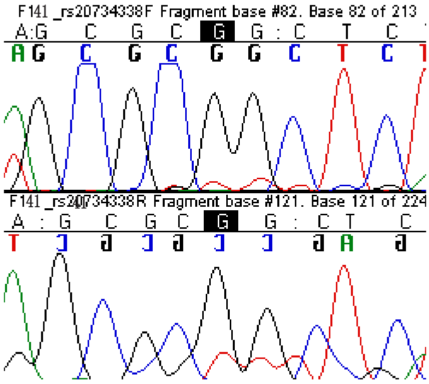	CAG	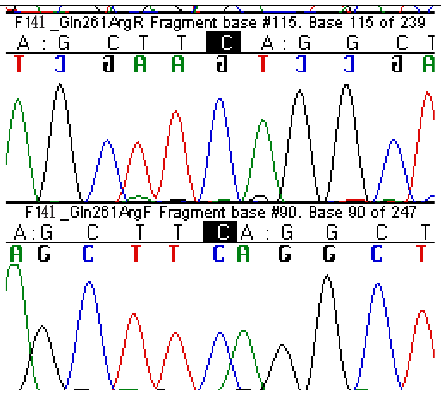
F104	G	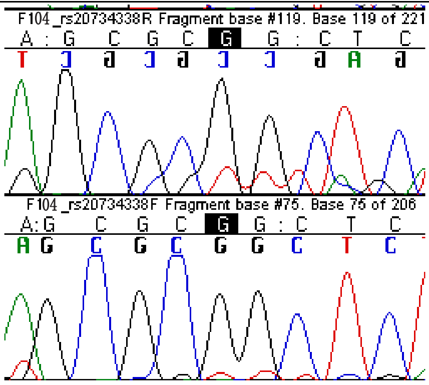	CAG/CGG	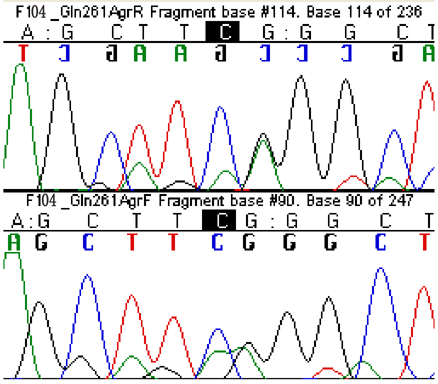
F106	G	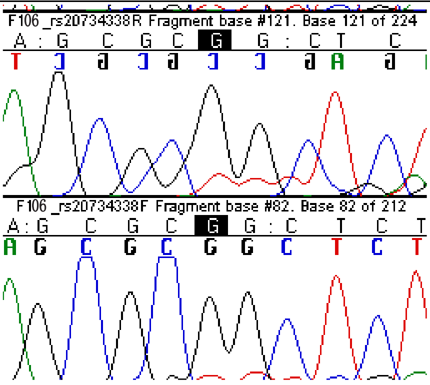	CAG/CGG	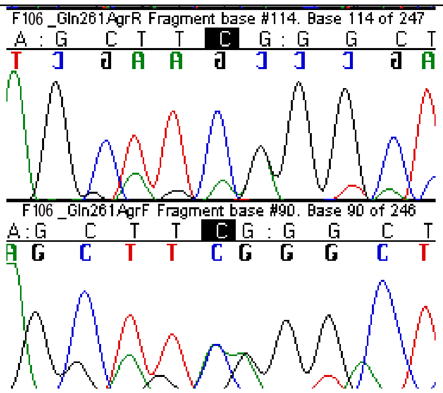

F107	G	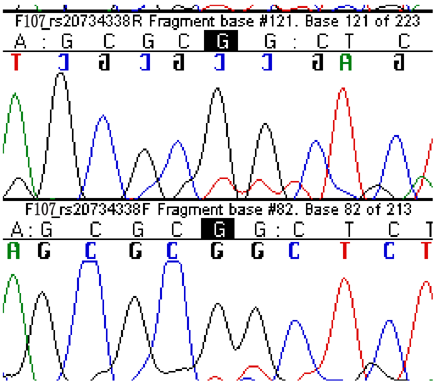	CGG	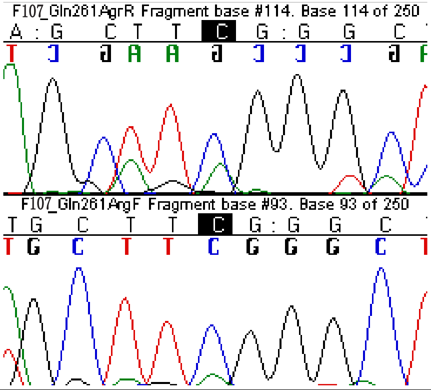
F138	G	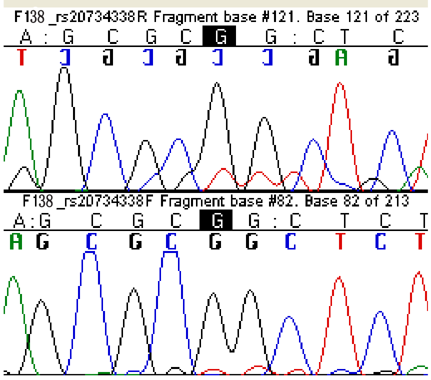	CAG/CGG	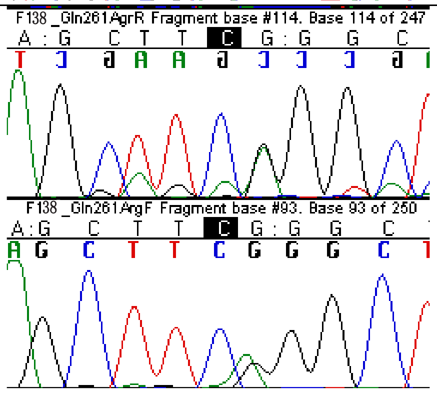
F120	G	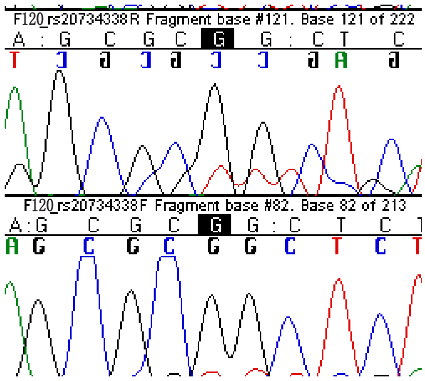	CGG	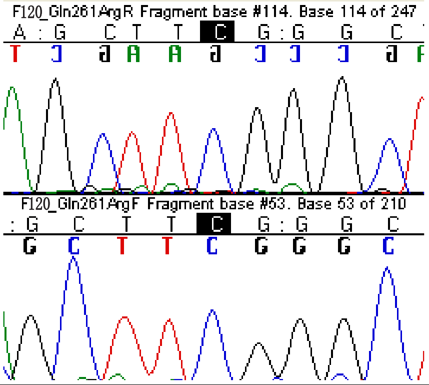
P103	G	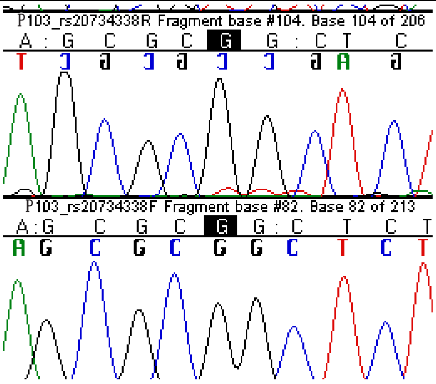	CAG/CGG	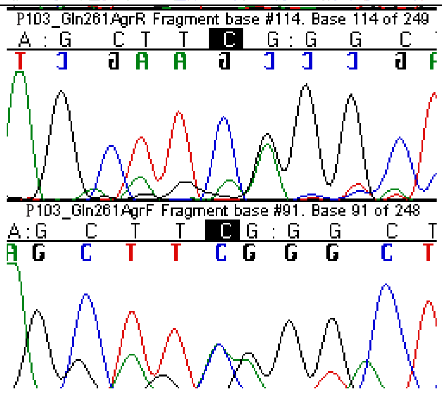

P67	G	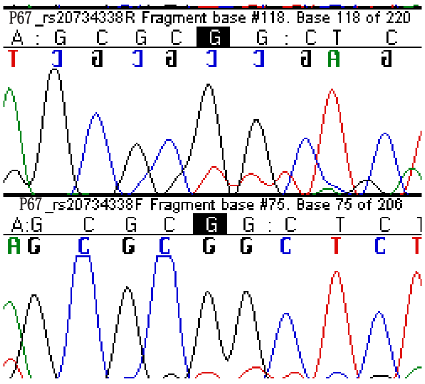	CGG	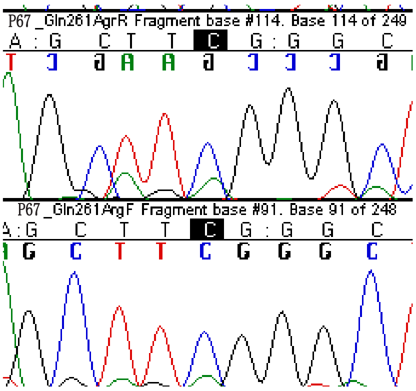
P68	G	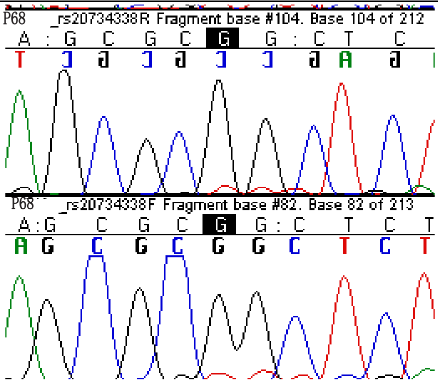	CGG	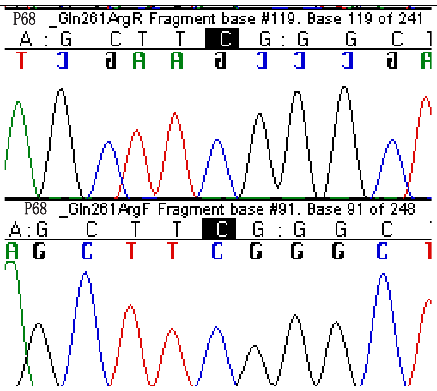
F81	G	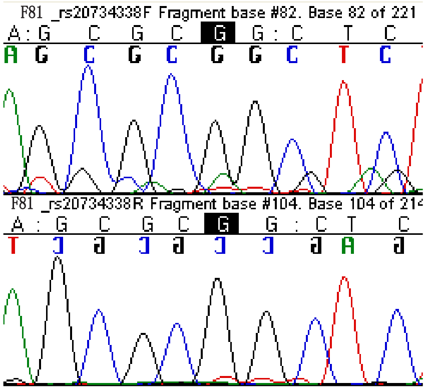	CAG/CGG	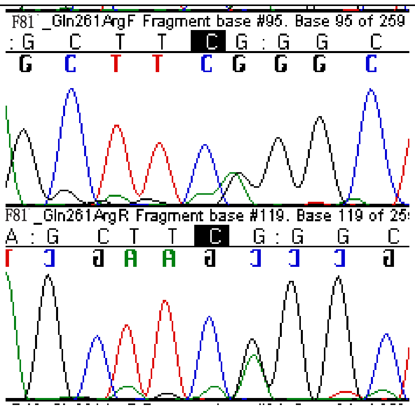
F08	G	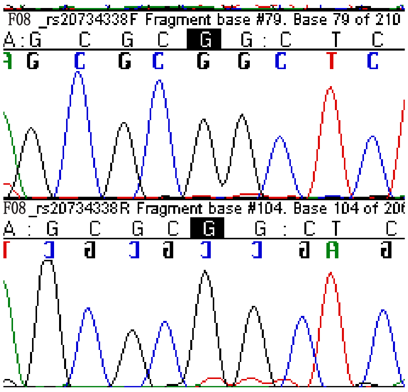	CAG/CGG	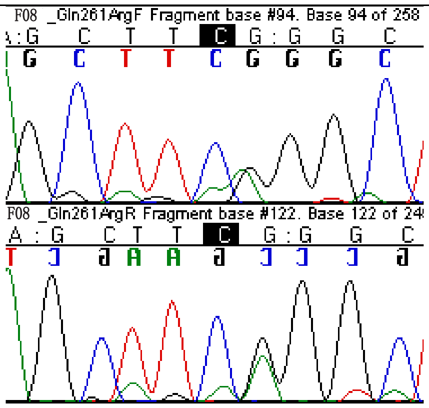

F25	G	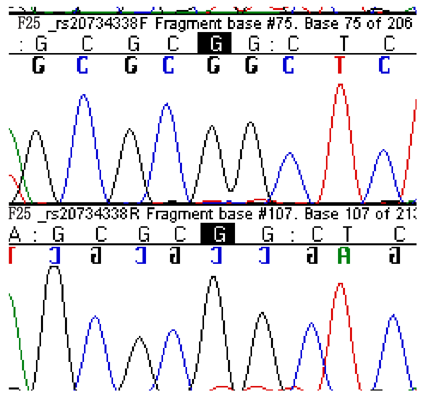	CGG	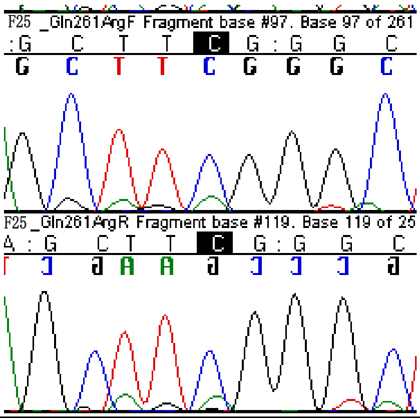
F52	G	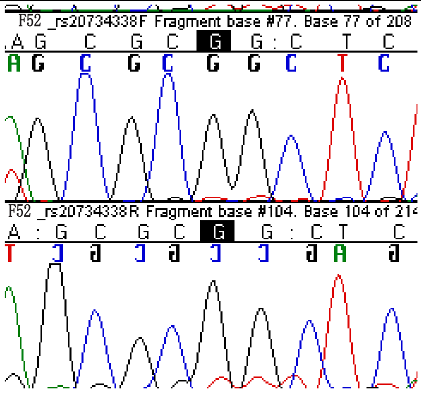	CAG/CGG	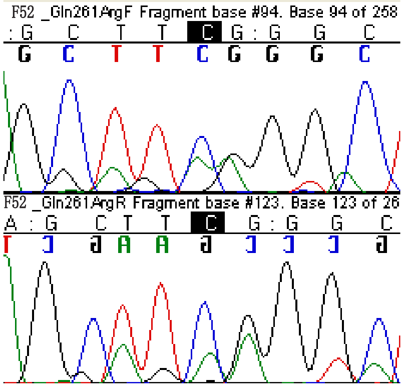
F14	G	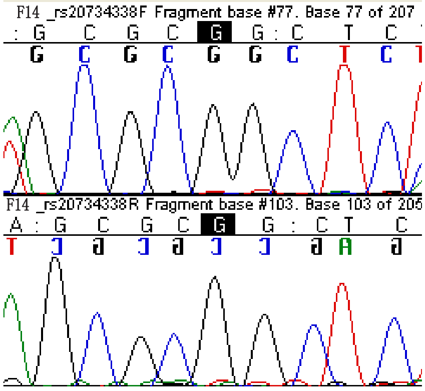	CGG	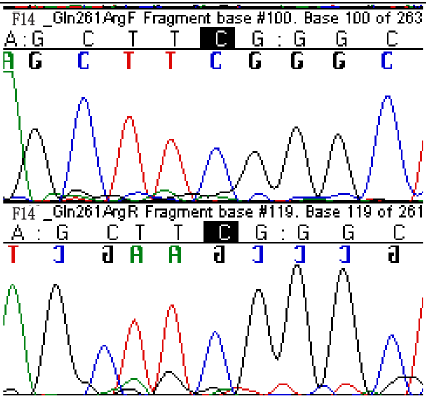
F63	G	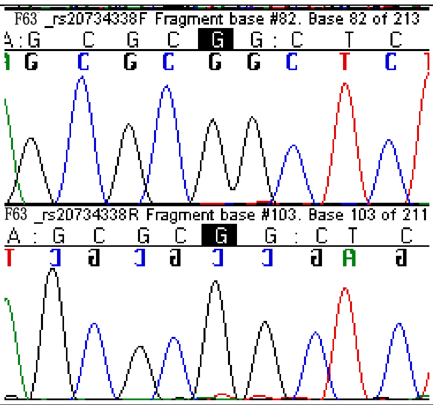	CGG	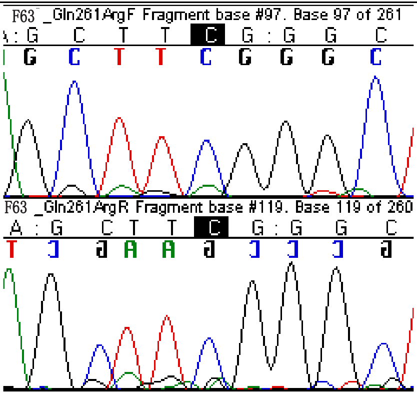

F87	G	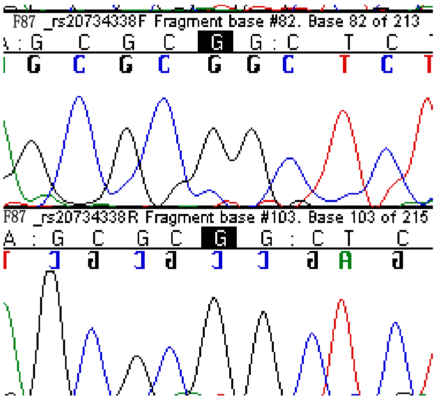	CAG/CGG	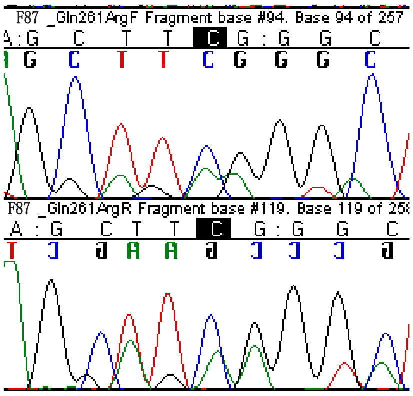
F13	G	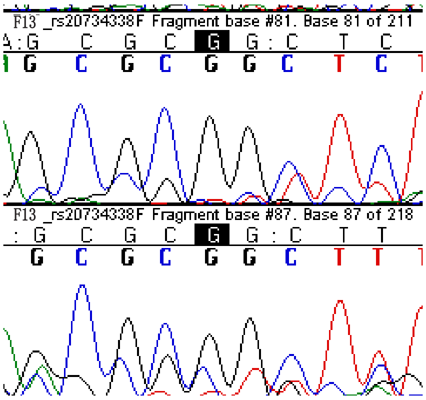	CAG/CGG	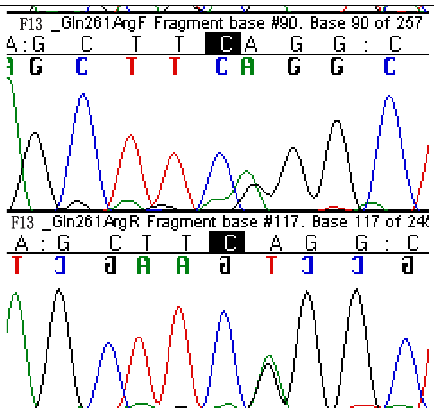
F45	G	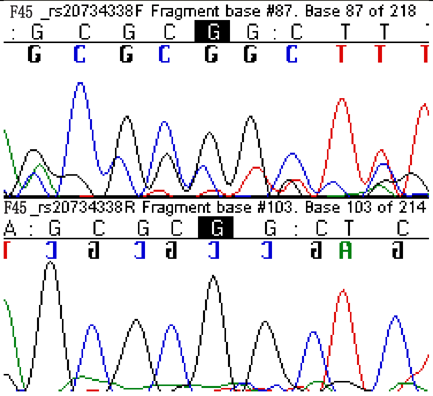	CGG	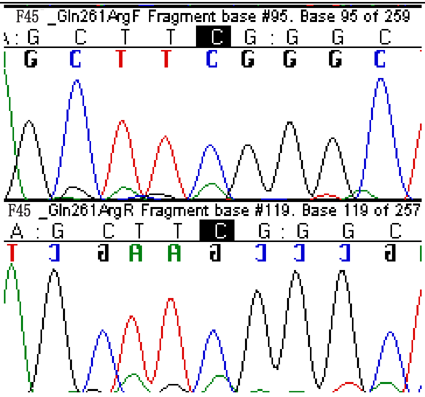
F09	G	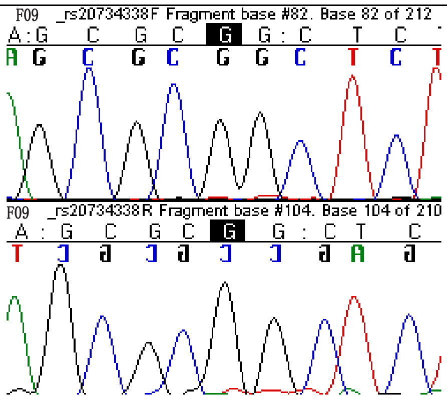	CGG	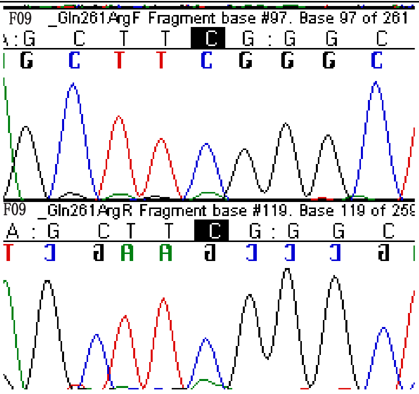

F05	G	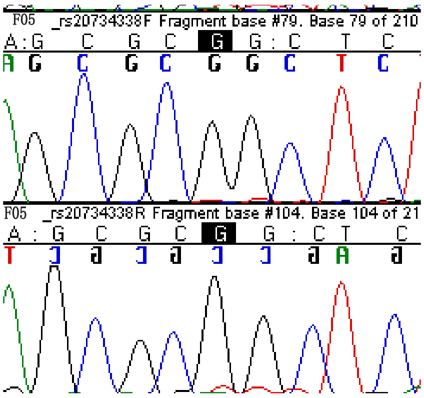	CGG	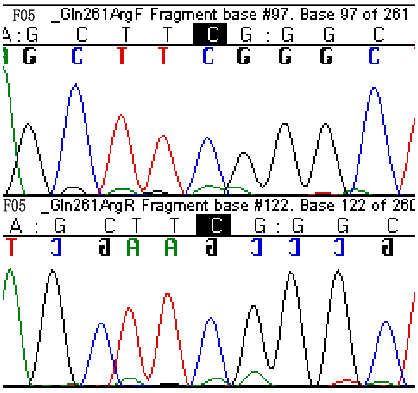
F22	G	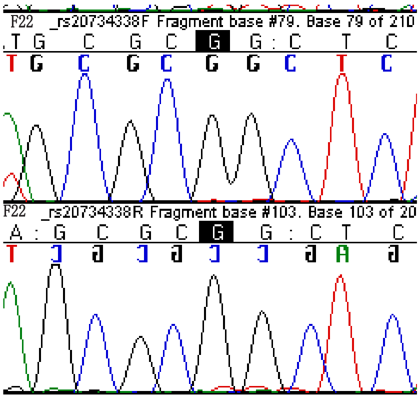	CAG/CGG	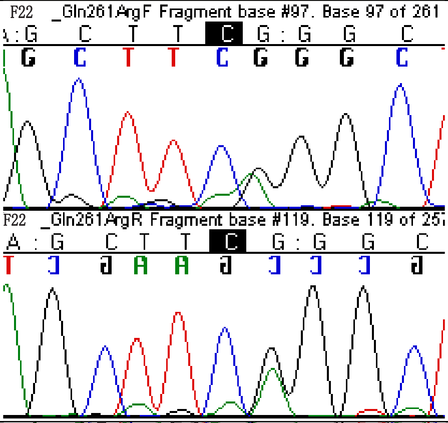
F96	G	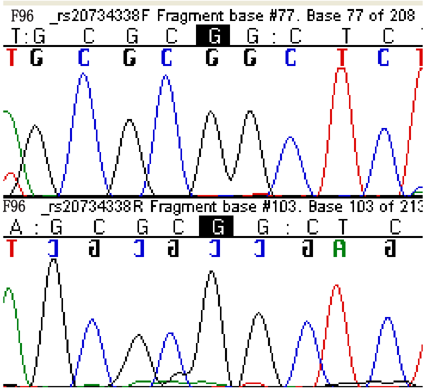	CGG	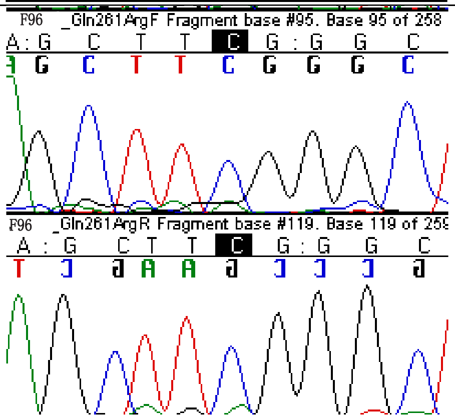
F78	G	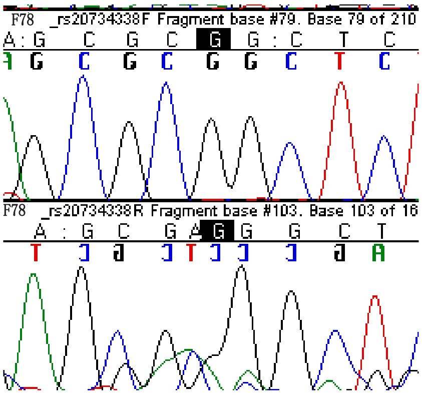	CAG/CGG	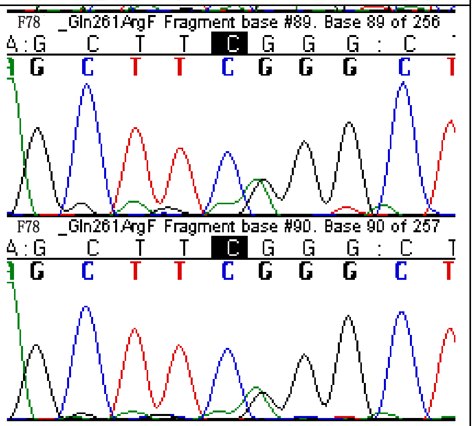

F84	G	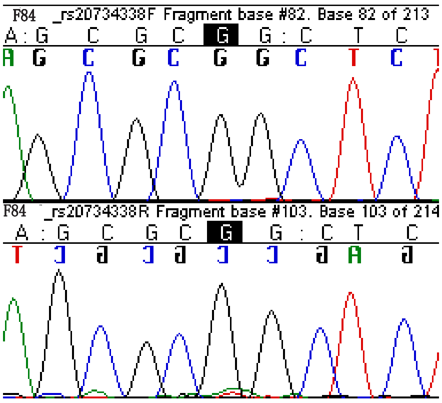	CAG/CGG	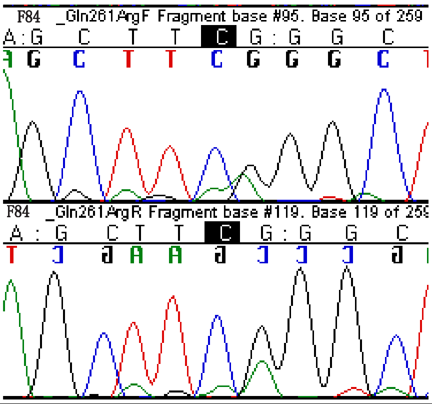
F82	G	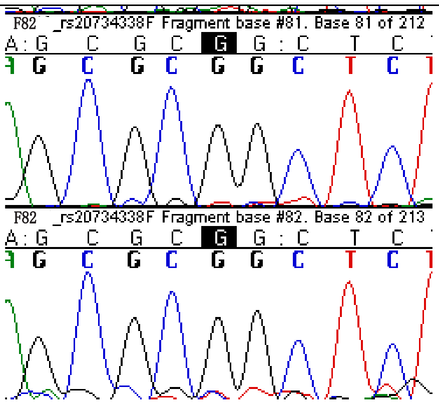	CGG	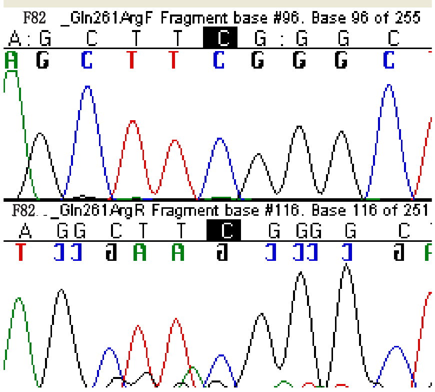
DA24	G	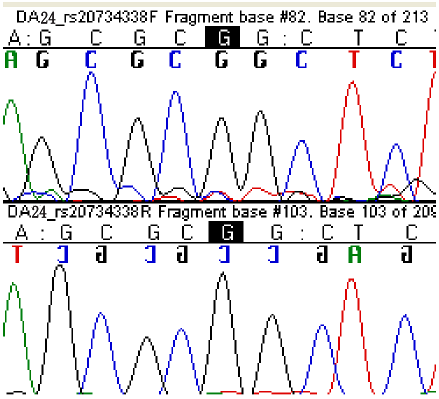	CAG/CGG	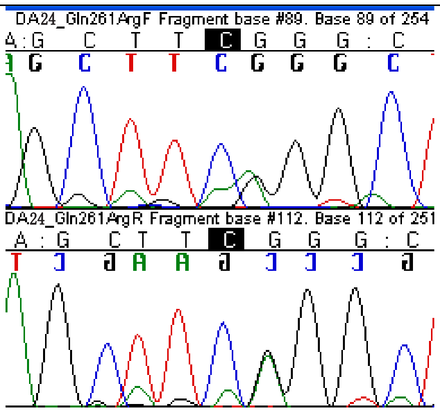
DA25	G	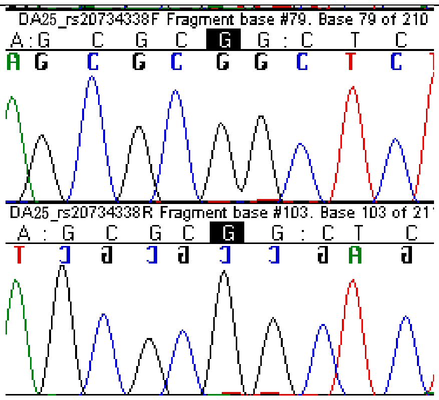	CGG	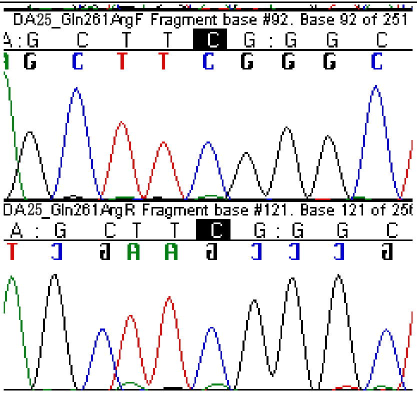

DA26	G	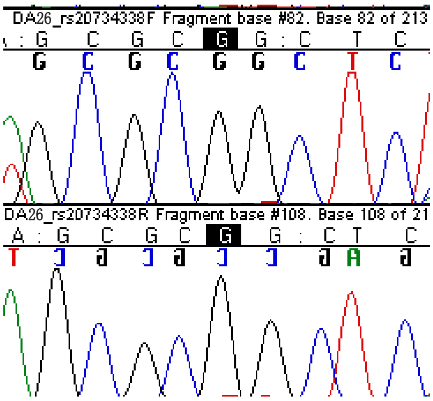	CGG	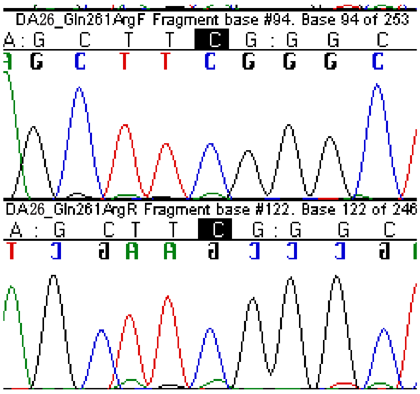
DA27	G	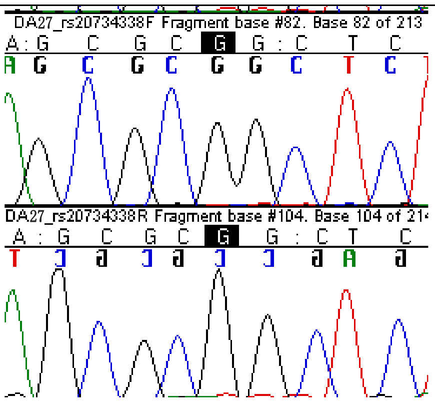	CAG/CGG	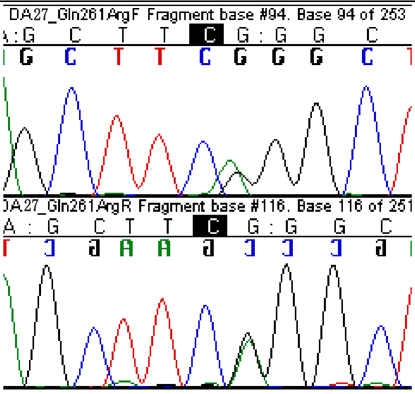
DA28	G	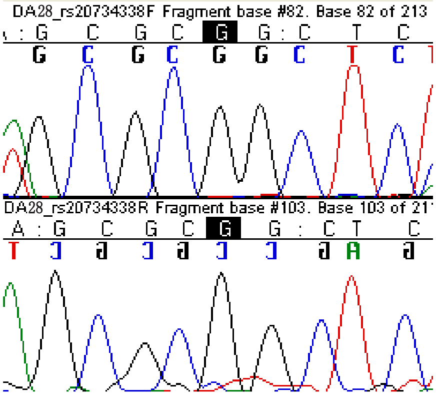	CAG/CGG	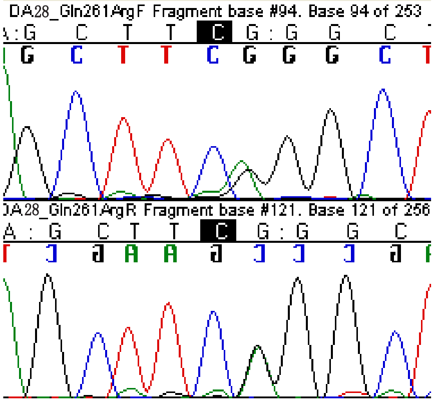
DA29	G	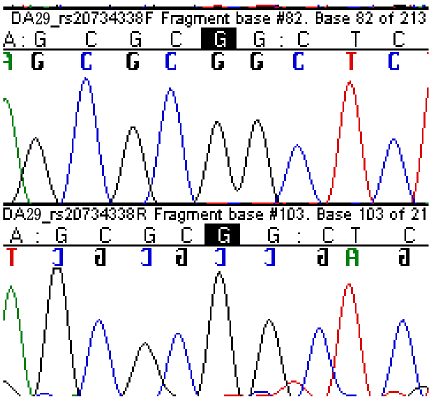	CAG/CGG	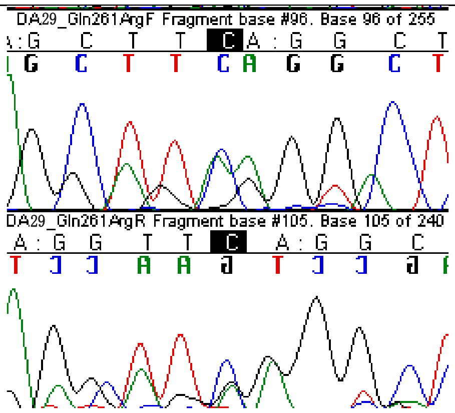

DA30	G	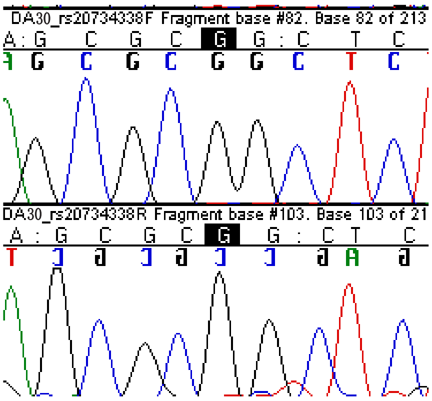 DA30_rs20734338F Fragment base #82. Base 82 of 213<br>DA30_rs20734338R Fragment base #103. Base 103 of 21	CGG	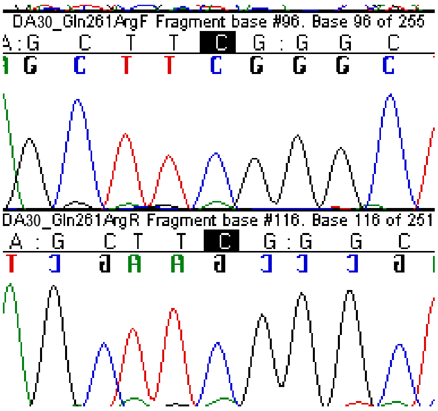 DA30_Gln261ArgF Fragment base #96. Base 96 of 255<br>DA30_Gln261ArgR Fragment base #116. Base 116 of 251
